# Filtering out the noise: metagenomic classifiers optimize ancient DNA mapping

**DOI:** 10.1093/bib/bbae646

**Published:** 2024-12-14

**Authors:** Shyamsundar Ravishankar, Vilma Perez, Roberta Davidson, Xavier Roca-Rada, Divon Lan, Yassine Souilmi, Bastien Llamas

**Affiliations:** Australian Centre for Ancient DNA (ACAD) and The Environment Institute, The School of Biological Sciences, University of Adelaide, Adelaide, SA, Australia; Australian Centre for Ancient DNA (ACAD) and The Environment Institute, The School of Biological Sciences, University of Adelaide, Adelaide, SA, Australia; Centre of Excellence for Australian Biodiversity and Heritage, University of Adelaide, Adelaide, SA, Australia; Australian Centre for Ancient DNA (ACAD) and The Environment Institute, The School of Biological Sciences, University of Adelaide, Adelaide, SA, Australia; Australian Centre for Ancient DNA (ACAD) and The Environment Institute, The School of Biological Sciences, University of Adelaide, Adelaide, SA, Australia; Faculty of Arts and Humanities, University of Coimbra, Coimbra, Portugal; Australian Centre for Ancient DNA (ACAD) and The Environment Institute, The School of Biological Sciences, University of Adelaide, Adelaide, SA, Australia; Genozip Limited, Hong Kong; Australian Centre for Ancient DNA (ACAD) and The Environment Institute, The School of Biological Sciences, University of Adelaide, Adelaide, SA, Australia; National Centre for Indigenous Genomics, Australian National University, Canberra, ACT, Australia; Indigenous Genomics, Telethon Kids Institute, Adelaide, SA, Australia; Australian Centre for Ancient DNA (ACAD) and The Environment Institute, The School of Biological Sciences, University of Adelaide, Adelaide, SA, Australia; Centre of Excellence for Australian Biodiversity and Heritage, University of Adelaide, Adelaide, SA, Australia; National Centre for Indigenous Genomics, Australian National University, Canberra, ACT, Australia; Indigenous Genomics, Telethon Kids Institute, Adelaide, SA, Australia

**Keywords:** ancient DNA, contamination, filtering, metagenomic classifiers, Kraken2

## Abstract

Contamination with exogenous DNA presents a significant challenge in ancient DNA (aDNA) studies of single organisms. Failure to address contamination from microbes, reagents, and present-day sources can impact the interpretation of results. Although field and laboratory protocols exist to limit contamination, there is still a need to accurately distinguish between endogenous and exogenous data computationally. Here, we propose a workflow to reduce exogenous contamination based on a metagenomic classifier. Unlike previous methods that relied exclusively on DNA sequencing reads mapping specificity to a single reference genome to remove contaminating reads, our approach uses *Kraken2*-based filtering before mapping to the reference genome. Using both simulated and empirical shotgun aDNA data, we show that this workflow presents a simple and efficient method that can be used in a wide range of computational environments—including personal machines. We propose strategies to build specific databases used to profile sequencing data that take into consideration available computational resources and prior knowledge about the target taxa and likely contaminants. Our workflow significantly reduces the overall computational resources required during the mapping process and reduces the total runtime by up to ~94%. The most significant impacts are observed in low endogenous samples. Importantly, contaminants that would map to the reference are filtered out using our strategy, reducing false positive alignments. We also show that our method results in a negligible loss of endogenous data with no measurable impact on downstream population genetics analyses.

## Graphical Abstract

**Graphical Abstract:**
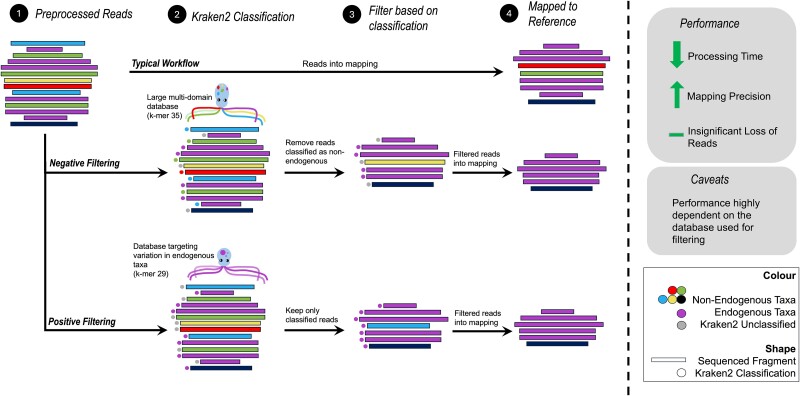

## Introduction

The field of paleogenomics relies on degraded ancient DNA (aDNA) molecules extracted from historic or prehistoric biological remains to study past environments and populations. Major progress in molecular methods to isolate and sequence aDNA has enabled the recovery of high-quality ancient genomes from many different species and sources [1–5]. However, contamination from modern and ancient exogenous sources remains a challenge that requires attention to improve the reliability and interpretative power of paleogenomic research.

Sample exposure to contaminating sources of DNA happens at various stages, including microorganisms and environmental DNA in the soil matrix; DNA from people who collected and handled samples in the field and/or museums and performed laboratory work [6]; and cross-contamination from different samples in the lab during DNA extraction and contamination from DNA sequences in reagents and consumables [7]. In short, a complex mixture of ancient and modern DNA contaminants continuously accumulates in and on the sample from the time of death of the organism up to laboratory work. As a result, endogenous DNA content is often outcompeted by exogenous DNA contaminants in sequenced data [8]. In the last decade, continuing improvements in laboratory protocols and established best practice guidelines have specifically addressed the issue of contamination in aDNA sequence data. From the excavation to library preparation and target enrichment, stringent measures are applied to minimize, identify, or discard exogenous DNA contaminants during sample handling and laboratory work [7].

Beyond these practical advances, sequencing data processing could assist with managing DNA contaminants. Currently, computational methods rely on the specificity of mapping shotgun sequences to a linear reference genome of, or closely related to, the species of interest [9]. However, spurious mapping of exogenous sequences to the target reference increases with decreasing fragment length and if the exogenous sequences come from a species closely related to the target reference. Recently, Feuerborn *et al.* [10] suggested the use of competitive mapping to remove human contamination from faunal aDNA datasets by mapping aDNA sequences against a composite reference sequence file containing both human and target reference genomes simultaneously. This technique is traditionally used in microbial genomics [11]. However, competitive mapping only considers a few sources of contamination and is not easily scalable to target multiple complex eukaryotic organisms due to increasing computational demand with larger composite reference genomes. Therefore, exogenous contamination not only complicates aDNA analysis but also intensifies the computational demands during sequence mapping. Postmapping filtering tools such as PMDtools [12] rely on the presence of aDNA damage misincorporations to remove contemporary contamination. However, not all endogenous aDNA reads present base misincorporations characteristic of aDNA damage, so this approach can lead to a large loss of endogenous sequences. Moreover, contaminating sequences from exogenous aDNA would not be removed by this approach. In short, efficient computational methods are lacking to not only identify but also efficiently remove contaminant DNA during the mapping process of aDNA datasets.

In recent years, the field of metagenomics has given rise to metagenomic classifiers capable of efficiently and accurately identifying diverse taxa in sequence data. These capabilities have shown potential in ancient metagenomic studies as well, although with specific caveats for each tool [13, 14]. We hypothesize that metagenomic classifiers offer an efficient approach to removing contaminant DNA by filtering them out before the mapping stage. As a result, we predict improved mapping accuracy and a significant reduction in computational resources needed, thereby making aDNA analysis more accurate and accessible across various computing platforms. A similar approach has been successfully applied to remove human patient DNA from clinical metagenomic data [15].

*Kraken2* [16] is a *k*-mer-based classifier initially designed to perform metagenomic analyses. Here, we propose an approach where *Kraken2* is used to identify and remove contaminating sequences from ancient DNA datasets of single organisms to accelerate the mapping process and improve mapping accuracy. We opted for a *k*-mer-based metagenomic classifier over alignment-based methods due to its faster processing speed [17], and we chose *Kraken2* because it presented the best balance between speed, database size, and classification accuracy compared to other metagenomic classifiers [18, 19], especially in ancient DNA contexts [20]. Using both simulated (human and dog) and empirical shotgun aDNA datasets, we show that this workflow presents a simple and efficient method that enables the removal of contaminating sequences from aDNA datasets with limited loss of endogenous DNA sequences while simultaneously reducing the overall computational resources needed during the mapping process as well as mitigating any potential errors introduced by spuriously mapping contaminant reads.

## Methods

### Data simulations

**Figure 1:**
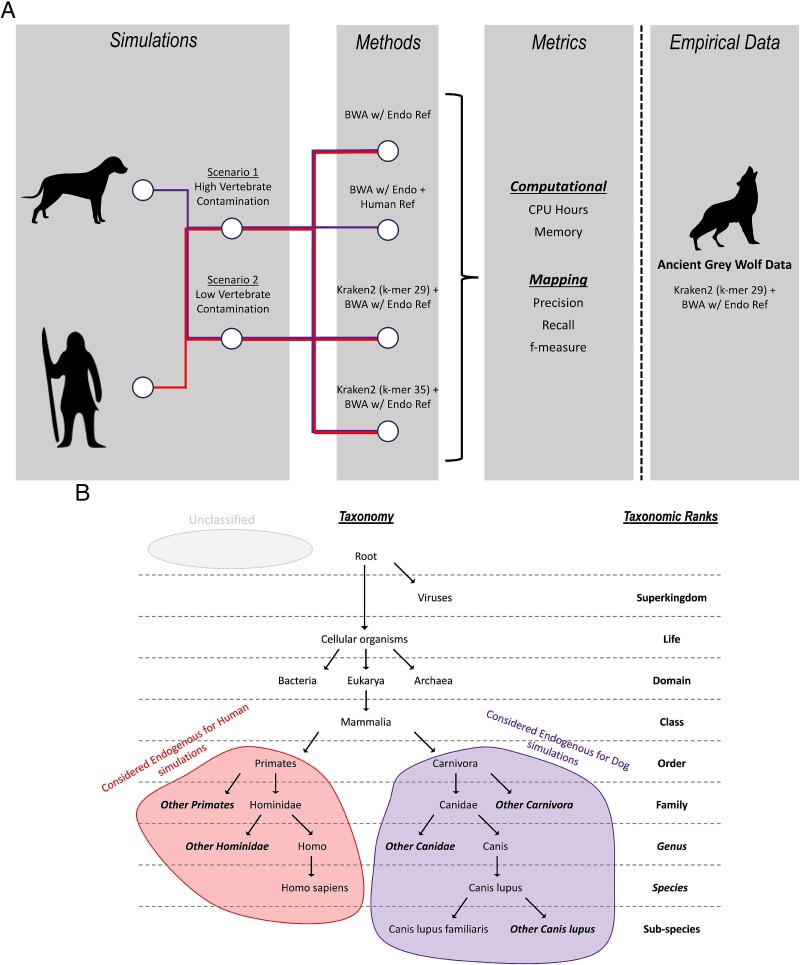
(A) Workflow of types of simulated data, different methods applied to the simulated data, and metrics collected. The best-performing method is applied to empirical data. (B) Ancient human reads classified at the order Primates or lower taxonomic ranks are considered endogenous reads and hence retained (red), similarly for ancient dog reads at the order Carnivora or lower (purple). Unclassified reads represented as grey are reads that could not be assigned a taxonomy.

We simulated ancient human (*Homo sapiens*) and dog (*Canis lupus familiaris*) shotgun sequencing datasets with varying levels of contamination (Fig. 1, Table S1) using *Gargammel 1.1.2* [21]. The human and dog reads, referred to as endogenous, were simulated from autosomal, sex, and mitochondrial contigs of *GRCh38.p14* (GCF_000001405.40) and *CanFam6* (GCF_000002285.5) genome assemblies, respectively. The exogenous contaminants consisted of modern human reads, represented by reads simulated from the same *GRCh38.p14* genome assembly; microbial contamination, represented by reads simulated from profiled microbial communities (Table S2) as presented by Seguin-Orlando *et al.* [22], including bacteria, viruses, and phages; and other contaminating reads representing common sources of contaminants found in aDNA datasets [23, 24]: sheep (*ARS-UI_Ramb_v2.0*; GCF_016772045.1), domestic cattle (*ARS-UCD1.3*; GCF_002263795.2), pig (*Sscrofa11.1*; GCF_000003025.6), goat (*ARS1.2*; GCF_001704415.2), and chicken (*GRCg6a*; GCF_000002315.6). Deamination profiles were simulated from the Loschbour individual in Lazaridis *et al.* [25] for the endogenous and microbial reads, simulating the damage profile of a single-stranded aDNA library partially treated with uracil–DNA–glycosylase (UDG) (Supplementary Fig. S1a). Finally, all the reads were simulated as paired-end Illumina HiSeqX reads with a read length of 75 bp and size distribution of the sequenced fragments simulated from the subset of a 45 000-year-old human sample from Siberia [26] (Supplementary Fig. S1b). Default *Gargammel* settings were used for the rest, including base quality distribution of the simulated bases and adding Illumina adapter sequences for fragments shorter than the read length (75 bp).

We simulated two scenarios with differing levels of modern human and other contamination in the dataset (Fig. 1A). For the ancient human dataset, the modern human portion was replaced with microbial sequences since a taxonomic classifier such as *Kraken2* will not be able to differentiate between modern and ancient reads from the same taxa. The endogenous proportion of the simulated reads ranged from 0.1% to 60%, with 20 million read pairs simulated for each (Table S1).

### Data processing

#### Preprocessing of simulated data

The simulated datasets were processed with *AdapterRemoval 2.3.2* [27] to trim adapters and merge paired-end reads with length and base quality filters ‘*--minlength 30*’ and ‘*--minquality 20*’, respectively [28]. Additionally, ‘*--qualitymax*’ was set to 64 to account for maximum read quality in the datasets simulated using *Gargammel*.

#### Data processing for baseline mapping performance

We mapped the merged reads using *bwa aln 0.7.17-r1188* [29] with the ‘*-n 0.01*’, ‘*-l 1024*’ and ‘*-o 2*’ ancient DNA parameters [30]. The human and dog datasets were mapped to *GRCh38.p14* and *CanFam6* genome assemblies, respectively. The mapped files were used to establish the baseline performance of *bwa aln* for all simulated endogenous levels.

#### Measuring baseline mapping performance

To measure the mapping performance, the reads were then categorized as ‘true positives’ ($\mathrm{TPs}$), ‘true negatives’ ($\mathrm{TNs}$), ‘false positives’ ($\mathrm{FPs}$), and ‘false negatives’ ($\mathrm{FNs}$) depending on the source taxonomy (endogenous or contamination) of the mapped read and its mapping quality to the reference genome (Table S3). These were used to calculate *precision, recall, and f-measure* to establish the baseline mapping performance [31].

$\mathrm{Precision}=\frac{\mathrm{TP}}{\mathrm{TP}+\mathrm{FP}}$ measures the proportion of correctly predicted positive instances out of all instances predicted as positive. In this context, precision quantifies the ratio of endogenous reads to all reads mapped to the reference.

$\mathrm{Recall}=\frac{\mathrm{TP}}{\mathrm{TP}+\mathrm{FN}}$ , also known as sensitivity or the true positive rate, measures the proportion of correctly identified positive instances from all positive instances in the dataset. In this context, recall quantifies the ratio of endogenous reads mapped to the reference to all endogenous reads in the dataset.

*f*-*measure* (or *f*-score): ${F}_1=2\cdotp \frac{\mathrm{Precision}\cdotp \mathrm{Recall}}{\mathrm{Precision}+\mathrm{Recall}}$ is the harmonic mean of *precision* and *recall*. It is typically used to quantify the overall performance of a classification model since optimizing only for precision or recall can have conflicting goals. A high ${F}_1$ value suggests accurate identification of positive instances while also minimizing false positives. In this context, ${F}_1$ quantifies a method’s ability to identify endogenous reads and contaminants in the dataset accurately.

#### Measuring the performance of competitive mapping

For the dog dataset, we also benchmarked competitive mapping using a composite reference with the *CanFam6* and *GRCh38.p14* assemblies as suggested by Feuerborn *et al.* [10], which has been shown to remove contemporary human contamination in ancient faunal datasets. We then mapped the reads using the composite reference and categorized reads according to Supplementary Table S4 to calculate *precision*, *recall*, and *f-measure*.

### Metagenomic classification

#### Measuring the performance of metagenomic filtering before mapping

We used *Kraken2* (v 2.1.3) [16], a *k*-mer-based method for taxonomic classification of sequence data. A *k*-mer refers to substrings of length *k* within a nucleotide sequence. *Kraken2* relies on the presence of exact *l*-mer (a subsequence of length *l*, where *l* ≤ *k*) matches between sequence data and a reference database containing known sequences and taxonomies to perform taxonomic classification.

To understand the effect of database composition on taxonomic classification, we created different databases that contained single species or sequences from multiple domains of life (Table 1). All databases were built with the default *k*-mer length of 35, as well as the *k*-mer length of 29. This choice of *k*-mer lengths is motivated by the fact that while a longer *k*-mer (i.e. 35) decreases the risk of false classification compared to a shorter *k*-mer (i.e. 29) [18], aDNA datasets generally use a cut-off of 30 bp for the minimal fragment length for mapping to a reference genome [32]. Therefore, a *k*-mer of 29 is likely more appropriate in the context of aDNA datasets to prevent a bias against very short reads. Finally, we also tested a publicly available database, *k2_nt_20230502* (https://benlangmead.github.io/aws-indexes/k2), which includes a larger collection of sequences across the three domains of life and viruses from the National Centre for Biotechnology Information (NCBI), inclusive of GenBank, RefSeq, Third Party Annotation (TPA), and Protein Data Bank (PDB).

**Table 1:** List of Databases used, their contents, k-mer length and memory required to run the database.

Name	Contents	*K*-mer	Memory (GB)
*k2_human*	Default Kraken Human Library	29	2.9
		35	3.9
*k2_microbes_human*	*k2_human + Default Kraken Libraries for archaea, viruses, bacteria, fungi, and protozoa*	29	35
		35	73
*k2_dog*	Sequences from CanFam6 (Dog)	29	2.6
		35	3.3
*k2_canis_lupus*	Sequences from CanFam6 (Dog), mCanLor1.2 (Grey Wolf) and ASM325472v2 (Dingo)	29	2.8
		35	3.7
*k2_canis_lupus + 722 g Variants*	*k2_canis_lupus + Consensus sequence of CanFam3.1 (Dog) genome with alternate alleles from 722 g project*	29	3.7
*k2_human_dog*	*k2_dog + Default Kraken Human Library*	29	5.1
		35	7.2
*k2_microbes_human_dog*	*k2_microbes_human + k2_dog*	29	35
		35	76
*k2_custom*	*k2_microbes_human_dog + Default Kraken Library for Plants + Sequences from ARS-UI_Ramb_v2.0 (sheep), ARS-UCD1.3 (cow), Sscrofa11.1 (pig), ARS1.2 (goat), GRCg6a (chicken), Bison_UMD1.0 (American Bison), panTro6 (chimpanzee), Kamilah_GGO_v0 (gorilla), ponAbe3 (orangutan), EquCab3.0 (horse), UM_NZW_1.0 (rabbit)*	29	58
		35	166
*k2_nt_20230502*	Very large collection, inclusive of GenBank, RefSeq, TPA, and PDB from Kraken2’s publicly available indexes	35	481

We evaluated databases based on their size and sensitivity when classifying endogenous sequences. The best-performing databases (see results) were used to filter reads from the simulated dataset before mapping. The filtered reads were then mapped to the reference with the same method as the baseline above. Finally, *precision*, *recall*, and ${F}_1$ were calculated after reads were categorized based on metagenomic filtering and mapping as per Tables S5 and S6.

#### Building Kraken2 databases

The *k*-mer 35 databases were built with the default options using genomes described in Table 1. The *k*-mer 29 database was built with options ‘*—kmer-len 29*’, ‘*—minimizer-len 24*’, and ‘*—minimizer-spaces 6*’ and included genomes as described in Table 1.

#### Kraken2 filtering

The *Kraken2* classifications were run with default parameters. A nextflow pipeline is available on github (https://github.com/shyama-mama/taxonomicfiltering) to perform filtering given a database and input reads.

### Empirical data

To validate our method with empirical data, we selected 10 ancient *Canis* samples from Bergström *et al*. [33] at similar endogenous proportions to the simulated data (Fig. 1A, Table S6). We mapped the data with *bwa aln* to the *CanFam6* reference genome following the parameters in the Data Processing for Baseline Mapping Performance section. We built a *Kraken2* database with a *k*-mer length set to 29 and composed of reference sequences from dog (*CanFam6*; GCF_000002285.5), grey wolf (*mCanLor1.2*; GCA_905319855.2), and dingo (*ASM325472v2*; GCF_003254725.2). Additionally, we also added a consensus *CanFam3.1* (GCF_000002285.3) reference using the alternate allele from bi-allelic single-nucleotide polymorphisms (SNPs) from 722 Canidae genomes from [34] into the database to minimize reference bias [35]. We used databases with and without the alternate allele information for filtering. We filtered the data by discarding any unclassified reads before mapping with *bwa aln* as above and compared both approaches: mapping only (Sample_BWA_) and filtering before mapping (Sample_Filt_).

To understand the effect of filtering on the data, for each sample, we extracted reads that met the following criteria: they mapped to the reference with a mapping quality score >20 when no premapping metagenomic filtering was performed, and they were also removed by *Kraken2* filtering when metagenomic filtering was performed. These reads were then classified using Nucleotide BLAST v2.14.1 [36]. We used the MEGAN v6.24.20 [37] suite of tools to get taxonomic abundances for each sample using the weighted lowest common ancestor (LCA) algorithm and following best-suited options for ancient DNA as suggested by Eisenhofer and Weyrich [13].

Since *Kraken2’s* taxonomic assignment relies on exact *l*-mer matches (where *l* ≤ *k*) to sequences in the database, there is a possibility to introduce reference bias when filtering relies on identifying endogenous sequences in the dataset. Hence, we used *f_4_* statistics from ADMIXTOOLS v7.0.2 [38] to assess if endogenous reads that were removed during *Kraken2* filtering introduced any bias in downstream analysis. The *f_4_* was performed with the configuration *f*_4_(Sample_Filt_, Sample_BWA_; Basenji01, Coyote01California), where Coyote01California and Basenji01, are coyote and dog, respectively, from Plassais *et al*. [34]. The effect of reference bias was tested for databases with and without alternate allele information. Pseudo-haploid genotype calls were generated for each sample using PileupCaller 1.5.2 (https://github.com/stschiff/sequenceTools) at polymorphic loci ascertained using heterozygous sites in the coyote genome [33] mapped to the *CanFam6* reference. To account for variability during random allele sampling, we replicated pseudo-haploid genotype calls three times per sample. We tested each sample for reference bias caused by filtering where a significant deviation from 0 in the positive direction indicates an introduced bias towards the reference due to filtering.

## Results

### Effect of database composition on taxonomic classifications

**Figure 2:**
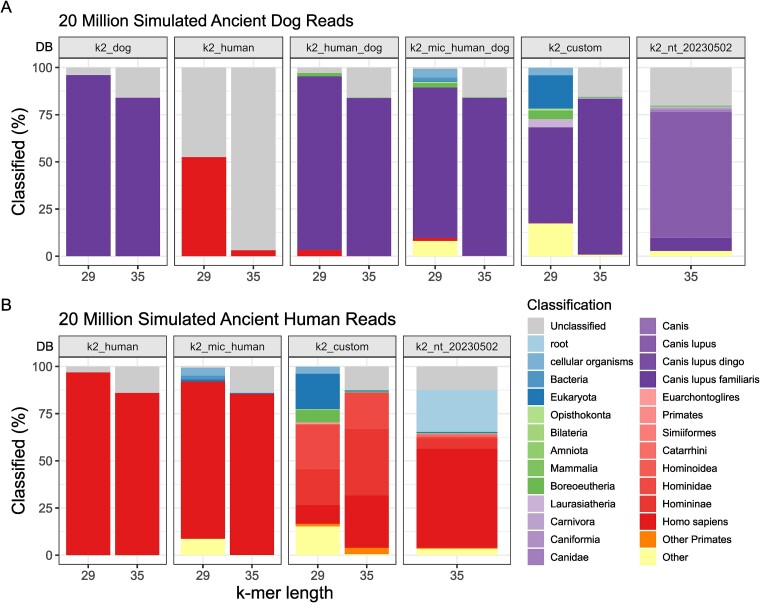
Impact of database choice on Kraken2 classification of (A) 20 million ancient dog reads, and (B) 20 million ancient human reads. The *x*-axis represents the *k*-mer length of the databases (DB; see Table 1 for descriptions) represented in the facets. The *y*-axis shows the proportion of reads classified as a particular taxonomy (colours).

Based on simulated ancient human and dog reads with no introduced contamination, we demonstrate that database composition significantly impacts classification accuracy (Fig. 2). Databases consisting of only a single genome exhibit a bias towards classifying sequences as belonging to that genome’s taxonomy. Conversely, more complex databases may compromise taxonomic resolution—i.e. the ability to classify sequences at the lowest specific taxonomic rank, as previously observed by Nasko *et al*. [39]. We observed a substantial reduction in dog reads misclassified as human from 3.17% to 0.12% and 52.52% to 3.34% for *k*-mer lengths 35 and 29, respectively, when using ‘*k2_human_dog*’, built from human and dog reference genomes, compared to ‘*k2_human*’, built with only the human reference (Fig. 2A). Using the combined database led to some reads classified as Boreoeutheria (0.26% and 1.77% for *k*-mer lengths 35 and 29, respectively), a classification rank that includes humans and dogs. Similarly, *Canis* sequences in ‘*k2_nt_20230502*’ led to dog reads classified primarily as *Canis* or *C. lupus* (Fig. 2A), and the presence of primate genomes in the ‘*k2_custom*’ database led to the classification of human reads as Hominidae or Homininae (Fig. 2B). We also note a substantial number of human reads classified as ‘root’ (22.22%) when using ‘*k2_nt_20230502*’. A ‘root’ classification is a result of the query sequence matching with viral and cellular organisms in the database (Fig. 1B). It is unclear if this is a result of contaminated viral sequences in the database or the presence of human endogenous retroviral sequences.

Databases built with *k*-mer length 29 decrease the size of the database and the number of unclassified reads—i.e. reads that could not be assigned a taxonomy (Fig. 1B). *Kraken2* assigns a classification to a read-only when the read contains unique *l*-mers (a subsequence of length *l*, where *l* ≤ *k*) that exactly match the *l*-mer sequences in the database [16]. Hence, databases built with a *k*-mer length of 29 assign taxonomies to shorter reads and reads with misincorporations due to damage better than the larger default *k*-mer length (Supplementary Fig. S2). However, this comes at the price of lower taxonomic resolution, with increased ancient dog and human reads classified as ‘cellular organism’ or ‘Eukaryota’ (21.38% and 22.31% for dog and human reads, respectively, using ‘*k2_custom*’), as well as increased false classifications as the simulated reads were misclassified as other vertebrate species in the database (>10% in dog reads using ‘*k2_custom*’).

​​​​.

### Benchmarking filtering before mapping

Filtering before mapping using metagenomic classifiers primarily aims to retain as many endogenous reads as possible while discarding contaminating sequences. However, sensible taxonomic classification is highly dependent on taxa represented in the database, regardless of database complexity. As we showed above, the rate of classification bias is driven by the *k*-mer length used to build the database, with lower specificity due to a shorter *k*-mer length leading to more aberrant classifications. Therefore, we investigated two strategies that take into account the strengths and drawbacks of different types of databases.

First, we applied filtering based on identifying contaminants using databases built with *k*-mer 35 and consisting of sequences from the target taxa and as many, as possible, contaminating genomes, to remove as many contaminating sequences as possible (negative filtering). We predict that this strategy is well suited for when the database is built with larger *k*-mer sizes, which show substantially lower false classification rates (Fig. 2).

Second, we applied filtering based on identifying endogenous reads using databases built with *k*-mer 29 and consisting of sequences from target taxa and genomes related to the target taxa to the family level, to retain as many endogenous reads as possible (positive filtering). We predict that this strategy is well suited for low *k*-mer sizes, which show higher classification rates at shorter read lengths (Supplementary Fig. S2).

The ancient human and dog reads were simulated as per the two scenarios in Fig. 1A and Table S1. Indeed, *Kraken2* is a metagenomic classifier, and it will not be able to differentiate between modern and ancient reads from the same species. Furthermore, while modern human contamination is a persistent problem in ancient human datasets, tools such as PMDtools have been shown to effectively remove them [12]. Subsequently, for the negative filtering strategy (removing contaminants), the simulated datasets were classified using the *‘k2_custom’* database, built with *k*-mer 35, to select reads classified at the order rank of the species of interest—Primates for human and Carnivora for dog—or remaining unclassified, before performing mapping with *bwa aln*. For the positive filtering strategy (retaining endogenous data), we used *‘k2_canis_lupus*’ and *‘k2_human*’ built with *k*-mer 29 for the dog and human datasets, respectively, and only mapped classified reads.

A positive outcome of filtering reads before mapping is shorter processing times. We see more than a 6-fold increase in processing speed (Supplementary Figs S3A and S4A, Tables S8 and S9). Here, processing time includes the runtime to classify, filter, and map reads when filtering is applied, as opposed to only mapping time when no filtering is applied. Interestingly, despite identical total read counts across all endogenous fractions, mapping took longer for data with high contamination from vertebrate sequences (i.e. Scenario 1 in Fig. 1 and Table S1) as opposed to data with low vertebrate contamination (i.e. Scenario 2 in Fig. 1 and Table S1), when mapped solely using *bwa aln*. This suggests that contaminants from taxa closely related to the target species’ reference disproportionately and negatively impact mapping time. Using a larger composite reference in competitive mapping further extended mapping times, up to 1.6-fold (Fig. S3A, Table S8). Combining competitive mapping with both *Kraken2* filtering strategies greatly improved mapping time, up to 4.4-fold and 6.8-fold faster when compared to single-reference and competitive mapping, respectively (Fig. S3A, Table S8).

By default, *Kraken2* databases are loaded into working memory and hence require, at minimum, free memory the same size as the database used. The ‘*k2_canis_lupus*’ (*k*-mer 29), and ‘*k2_human*’ (*k*-mer 29) databases are relatively small at 2.6 and 2.9 GB, respectively, making it possible to filter data even on personal machines. In contrast, the *‘k2_custom’* (*k*-mer 35) database is substantially larger, at 166 GB, and is better suited for high-performance computing systems. Importantly, premapping filtering, regardless of the filtering strategy, greatly reduces the read volume for highly contaminated samples (Tables S8 and S9), which, in turn, facilitates a more efficient mapping process, sparing computational resources.

**Figure 3:**
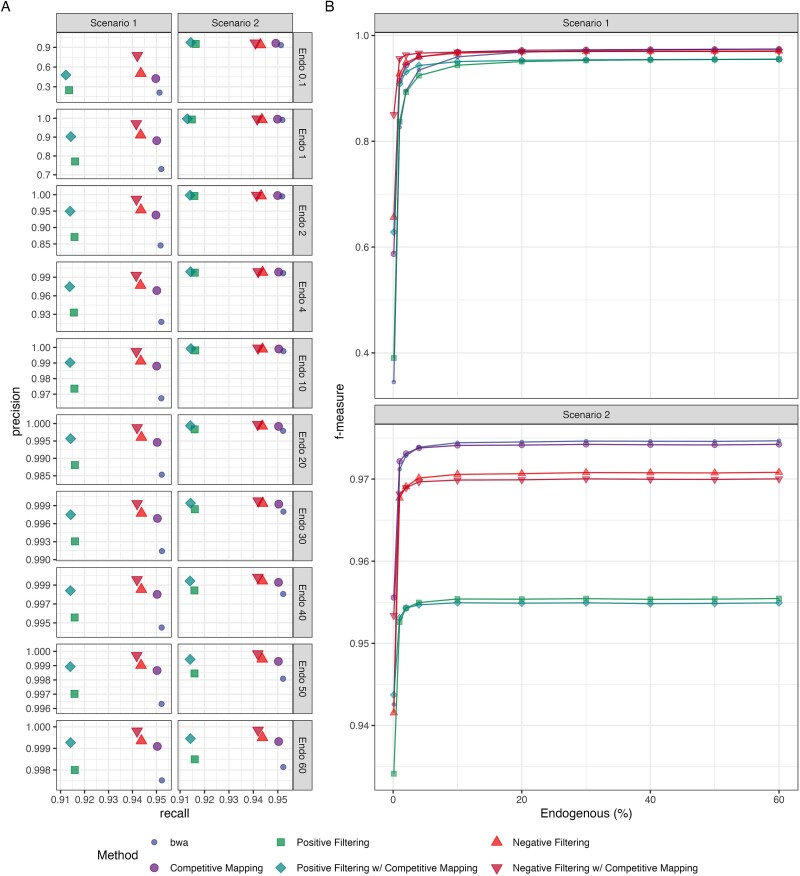
Precision and recall (A) and f-measure (B) of the six methods—bwa mapping to a single (colour: blue & shape: small circle) and composite dog and human reference (competitive mapping; colour: purple & shape: large circle), mapping only reads classified as Carnivora and unclassified reads by the ‘k2_custom’ database to a single (negative filtering; colour: light red & shape: triangle) and composite reference (negative filtering w/ competitive mapping; colour: dark red & shape: upside-down triangle), mapping only reads classified by ‘k2_canis_lupus_kmer29’ database to a single (positive filtering; colour: light green & shape: square) and composite reference (positive filtering w/ competitive mapping; colour: dark green & shape: diamond)—for the simulated ancient dog genome. Reads were filtered with MapQ >20 postmapping.

Across all endogenous proportions tested, both *Kraken2* filtering strategies consistently yielded more precise mapping compared to *bwa aln* mapping alone for both the high and low vertebrate contaminations (Fig. 3A and Supplementary Fig. S5A; Tables S8 and S9). Competitive mapping was also more precise compared to *bwa aln* mapping to a single reference, indicating it can accurately remove human contaminants from the faunal data, albeit with the longest run time. Since competitive mapping was only used to remove human contamination, other microbial and vertebrate contamination introduced remained in the mappings (Figs S10–S13). Combining competitive mapping with *Kraken2* filtering improved mapping accuracy beyond what either method achieved alone while significantly reducing the processing time required for competitive mapping (Figs 3A and S5A).

We summarized the *precision* and *recall* of each method by read length (Figs S6–S9). Both competitive mapping and positive filtering showed the largest precision increase for shorter fragments (30–40 bp) compared to longer fragments when benchmarked against *bwa aln* mapping to a single reference. Interestingly, negative filtering achieved the highest precision improvement with fragments 41–50 bp in length. This is due to the *k*-mer 35 database used for negative filtering; shorter fragments <35 bp cannot be classified with this *k*-mer length. Additionally, since negative filtering retains unclassified reads for mapping with *bwa aln* to maximize endogenous read retention, the precision increase is less pronounced for 30–40 bp fragments than for 41–50 bp fragments.

We observe a greater loss of endogenous reads when applying both Kraken2 filtering strategies compared to mapping directly to the reference, with losses up to 0.99%, and 3.8% for negative and positive filtering, respectively (Tables S8 and S9). For negative filtering, this lower recall stems from endogenous reads misclassified as other taxa, a known limitation of *Kraken2*’s probabilistic compact hash table, which can lead to false classifications. This loss is more pronounced with fragments 41–50 bp in length. In positive filtering, short fragments (<50 bp) were filtered out, as the *k*-mer 29 database struggled to classify these reads, despite the shorter *k*-mer length (Figs S6–S9). Despite this loss of endogenous reads, the resulting precision increase from filtering improved f-measures for low endogenous samples with high vertebrate contamination (Scenario 1). In contrast, this same loss of endogenous reads lowered f-measure for samples with more endogenous data or less vertebrate contamination (Scenario 2) when compared to *bwa aln* mapping to a single reference (Figs 3B and S5B). Competitive mapping, in comparison, showed the lowest loss of endogenous reads across all fragment lengths and endogenous fractions. Consequently, combining *Kraken2* filtering with competitive mapping improved the *f*-measure beyond what Kraken2 filtering achieved individually.

### Filtering empirical data

**Figure 4:**
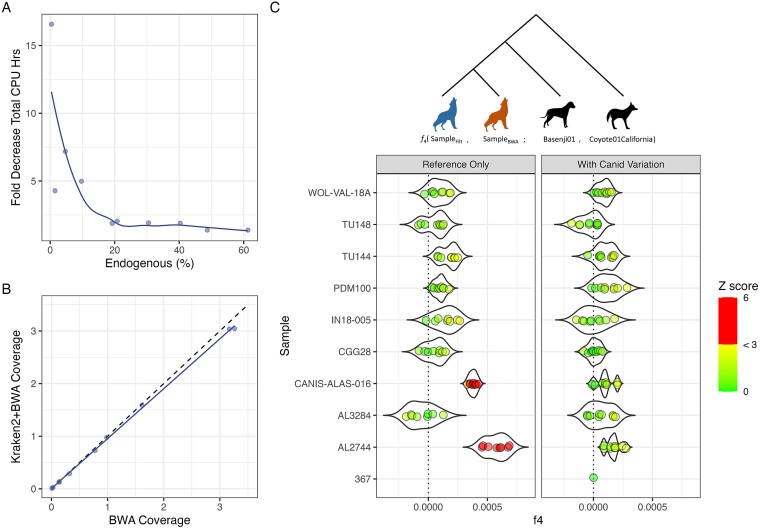
(A) Kraken2 + bwa aln filtering and mapping speed, normalized to bwa aln mapping only speed; (B) coverage difference between mapping only and filtering before mapping; and (C) observed f4 statistics of the configuration f4(SampleFilt (blue), SampleBWA (brown); Basenji01, Coyote01California) from pseudo-haploid genotypes. Multiple points indicate replicate pseudo-haploid calls to account for variability introduced by random pseudo-haploidization. The points are coloured by |Z| score. |Z| values below 3 are on a green-to-yellow gradient. |Z| values above 3 are denoted with red. Filtering using only the reference genome (left panel) led to samples CANIS-ALAS-016 and AL2744 being significantly biased (Z > 3) towards the reference. Adding Canid variation from the 722 g project (right panel) shows a nonsignificant deviation (|Z| < 3) from 0 for all samples.

Finally, we mapped classified reads from 10 ancient grey wolf samples from Bergström *et al*. [33] using the positive filtering strategy with the ‘*k2_canis_lupus*’ database built with *k*-mer 29 and including biallelic SNPs from 722 Canidae genomes [34]. We see up to a 16-fold increase in processing speed (sample 367 with 0.4% endogenous DNA) when filtering with *Kraken2* (Fig. 4A, Supplementary Table S10). The performance boost was observed across samples, with even those having a higher endogenous fraction of 20% and 50% or more experiencing a 2-fold and 1.3-fold increase in processing speeds, respectively. In line with our simulated results, we observed some loss of endogenous data (Fig. 4B, Supplementary Table S10).

Reads that were mapped to the reference with a mapping quality score above 20 when no premapping metagenomic filtering was performed but were filtered out during metagenomic filtering were assigned taxonomies using BLAST and LCA algorithms in MEGAN. Most of these reads could not be assigned a taxonomy (41.80%–76.55%), with the majority of the classified reads assigned to the Order Carnivora (> 93% of classified reads). Of note, all samples had reads classified at the order Primates (0.03%–0.70%). Competitive mapping against *CanFam6* and *GRCh38.p14* references saw the reads map preferentially to the human reference. Since these reads were originally also mapped to the *CanFam6* reference, we were able to compare the damage profiles of these reads mapping to the two references independently. Six out of the 10 samples (367, IN18-005, TU114, TU148, WOL-VAL-18A, and CANIS-ALAS-016) showed spurious C-to-T and G-to-A misincorporations when mapped to the dog reference, whereas no such misincorporations were observed when mapped to the human reference, suggesting these reads are modern human contamination (Supplementary Fig. S14). We also observed up to 3.58%, 0.82%, 0.27%, 0.26%, and 0.25% reads classified as bacteria, Artiodactyla, Rodentia, Chiroptera, and Lepidoptera, respectively (Supplementary Table S11). The authenticity of these classifications was not determined.

We see no significant bias (|Z| < 3) introduced by filtering with *Kraken2* when the database used to select endogenous reads contains variation information for Canids (Fig. 4C). However, it should be noted that samples CANIS-ALAS-016 and AL2744 observed low but significant reference bias when the *Kraken2* database used to identify endogenous reads did not contain the alternate allele information (Fig. 4C, Supplementary Tables S12 and S13).

## Discussion

Our study presents evidence that premapping filtering using *Kraken2* not only optimizes the usage of computational resources by greatly reducing mapping time but also improves the precision of mapped aDNA reads. This is particularly evident in datasets involving samples with very low levels of endogenous sequences and high contamination from sequences closely related to the target species. By implementing a positive filtering strategy to retain putative endogenous reads using a *Kraken2* database built with *k*-mer 29 consisting of reference and alternate sequences, and genomes from closely related taxa to the species of interest, we achieve a streamlined process that is resource-efficient and suitable for a wide array of computational environments, including personal machines due to the database requiring <5 GB of memory to run.

A more thorough approach is negative filtering to remove putative contaminants. This strategy utilizes a comprehensive database built with *k*-mer 35 and encompassing a broad spectrum of contaminants and affords enhanced precision and recall. The increased memory requirements for a larger database means this approach is more suitable for researchers who have access to high-performance computing resources. However, the feasibility of this strategy depends on how well the database represents the full array of potential contaminants in a particular dataset because the filtering depends on how many contaminants can be classified and hence removed. Identifying environmental microbes is a limitation of empirical data since most reference databases focus on human pathogens or microbial species that are of interest to humans [40–46]. However, in recent years, ancient and modern environmental microbiomes have increasingly been characterized [47, 48]. The development of resources like the Genomic Taxonomy Database [49] is helping bridge the gap between genomics and microbial taxonomy, improving microbial characterization across diverse environments, including contamination present in ancient datasets.

Our findings suggest that the choice of strategy should be guided by the available resources and specific priorities. The loss of some negligible amounts of endogenous data is an inherent limitation of the *Kraken2* filtering approach, but this trade-off is balanced by gains in mapping efficiency and precision. Endogenous read loss occurs either through misclassifications—more common in negative filtering—or through unclassified reads in the positive filtering strategy. Misclassifications stem from the probabilistic compact hash table used by *Kraken2*, which, though memory-efficient compared to a standard hash table, sacrifices some specificity and accuracy [16]. Likewise, unclassified reads in positive filtering are also because of this, with shorter, damaged DNA fragments being especially affected (Fig. S2).

We also caution against filtering by identifying endogenous reads when studying extinct species for which no reference genomic resources exist, as it might impact the retrieval of endogenous reads and cause the reads to be biased towards the reference alleles in the database. For well-studied species such as humans and dogs, we propose adding alternate allele information from large genomic studies to better capture variation in the sequence data. Furthermore, the processing time of ancient hominin genomes enriched with the 1240 k SNP panel [50] could be greatly reduced if a database with the human reference and the expected alternate allele information captured by the panel were used to select human reads before mapping. Our findings coincide with the increasing use of pangenomic approaches for genomic analysis, such as adding alternate allele information to reduce reference bias during mapping [51], building databases from sequences from pangenomic projects for improved host removal from clinical metagenomic data [15], and novel tools such as Euka [52] that use pangenomic graphs for metagenomic classification—although currently, Euka databases are restricted to mitochondrial genomes of tetrapods and arthropods.

We also highlight that using databases with microbial and human sequences to classify human DNA can lead to some human reads being classified at the kingdom or domain rank as previously observed [18] because some microbial sequences are contaminated with human DNA. It is thus imperative to benchmark databases against the project’s objectives to mitigate these issues and their effect on data interpretation, which aligns with the growing body of literature that underscores the importance of benchmarking metagenomic classifiers in different contexts [13, 14, 18–20, 53, 54]. In recent years, there have been increased efforts in characterizing and removing contaminated sequences in reference databases to reduce erroneous interpretations of metagenomic datasets [55–57].

We propose that an approach that includes classification-based filtering has the potential to refine data processing and improve overall mapping data quality. We anticipate that continued improvements in metagenomic classifiers and reference databases that can identify environmental taxa will result in increased accuracy of our proposed filtering approaches and reduce data loss, paving the way for more precise reconstructions of ancient genomes.

**Key Points**

- Contamination is a major challenge in paleogenomics. Computational methods are essential to distinguish between endogenous and contaminant sequences.
- We propose a new workflow relying on a metagenomic classifier to filter out contaminants prior to aligning sequences to a reference sequence.
- We provide clear strategies to build the reference database and finetune the parameters to optimize the classification.
- Our workflow significantly reduces the computational resources and overall runtime while improving mapping precision and downstream analyses.

## Supplementary Material

BIB_supplementary_document_revised_bbae646

BIB_supplementary_tables_bbae646

## Data Availability

All reference genomes used and their associated accession numbers can be found in Supplementary Table S7. The publicly available *Kraken2* nucleotide database, *k2_nt_20230502*, was downloaded from https://benlangmead.github.io/aws-indexes/k2 and the 2023/05/02 version used for the manuscript. The VCF file for the National Human Genome Research Institute (NHGRI) Dog Genome Project was downloaded from the National Institutes of Health (NIH) (https://research.nhgri.nih.gov/dog_genome/downloads/datasets/WGS/).

## Code availability

The filtering workflow is available at https://github.com/shyama-mama/taxonomicfiltering. Other code for the manuscript can be found in https://github.com/shyama-mama/taxonomicfilteringmanuscript.
