# Supplementary material for: Filtering out the noise: metagenomic classifiers optimize ancient DNA mapping
Source: Brief Bioinform. 2024 Dec 14;26(1):bbae646. doi: 10.1093/bib/bbae646 (PMC11646131; doi:10.1093/bib/bbae646)
Supplement: BIB_supplementary_document_revised_bbae646 [file bib_supplementary_document_revised_bbae646.docx]

## Supplementary Information for

Filtering out the noise: Metagenomic classifiers optimise ancient DNA mapping

*Shyamsundar Ravishankar^1,+^, Vilma Perez^1,2^, Roberta Davidson^1^, Xavier Roca-Rada^1,3^, Divon Lan^1,4^, Yassine Souilmi^1,5,6^*, and Bastien Llamas^1,2,5,6,*,+^*

^1^ Australian Centre for Ancient DNA (ACAD) and The Environment Institute, The School of Biological Sciences, The University of Adelaide, Adelaide, South Australia

^2^ Centre of Excellence for Australian Biodiversity and Heritage, University of Adelaide, Adelaide, SA 5005, Australia

^3^ Faculty of Arts and Humanities, University of Coimbra, Coimbra, Portugal

^4^ Genozip Limited, Hong Kong

^5^ National Centre for Indigenous Genomics, Australian National University, Canberra, ACT 0200, Australia

^6^ Indigenous Genomics, Telethon Kids Institute, Adelaide, SA 5000, Australia

* Equal contribution

+ Corresponding Authors

## Document Contents

Supplementary Figures S1-S14

Supplementary Tables S1-S6

## Supplementary Figures


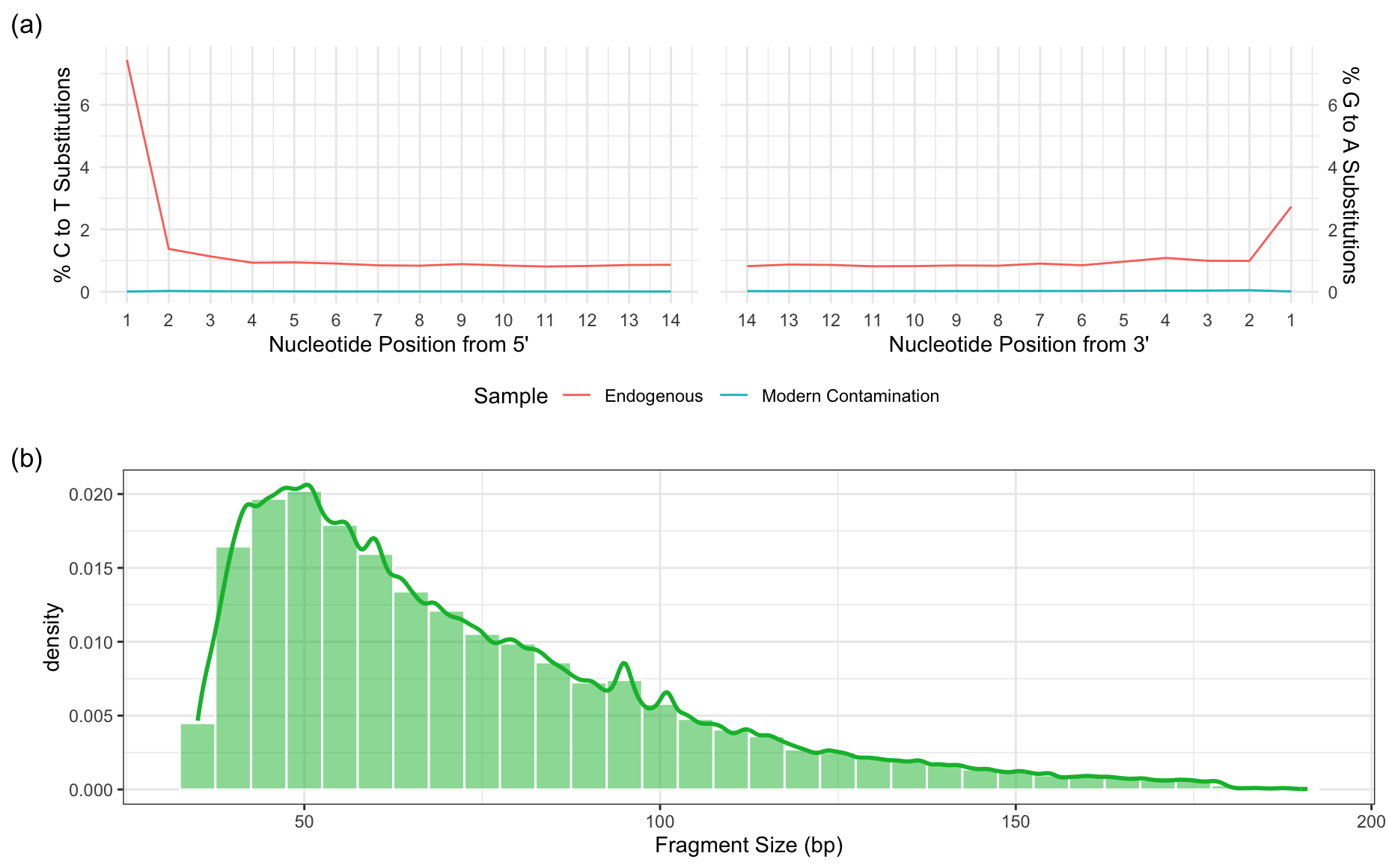


**Figure S1:** (A) Ancient DNA damage profile that was applied to endogenous and microbial reads (red) and no damage profile for modern human and reagent contamination (blue); (B) Fragment size distribution of all simulated reads.

**
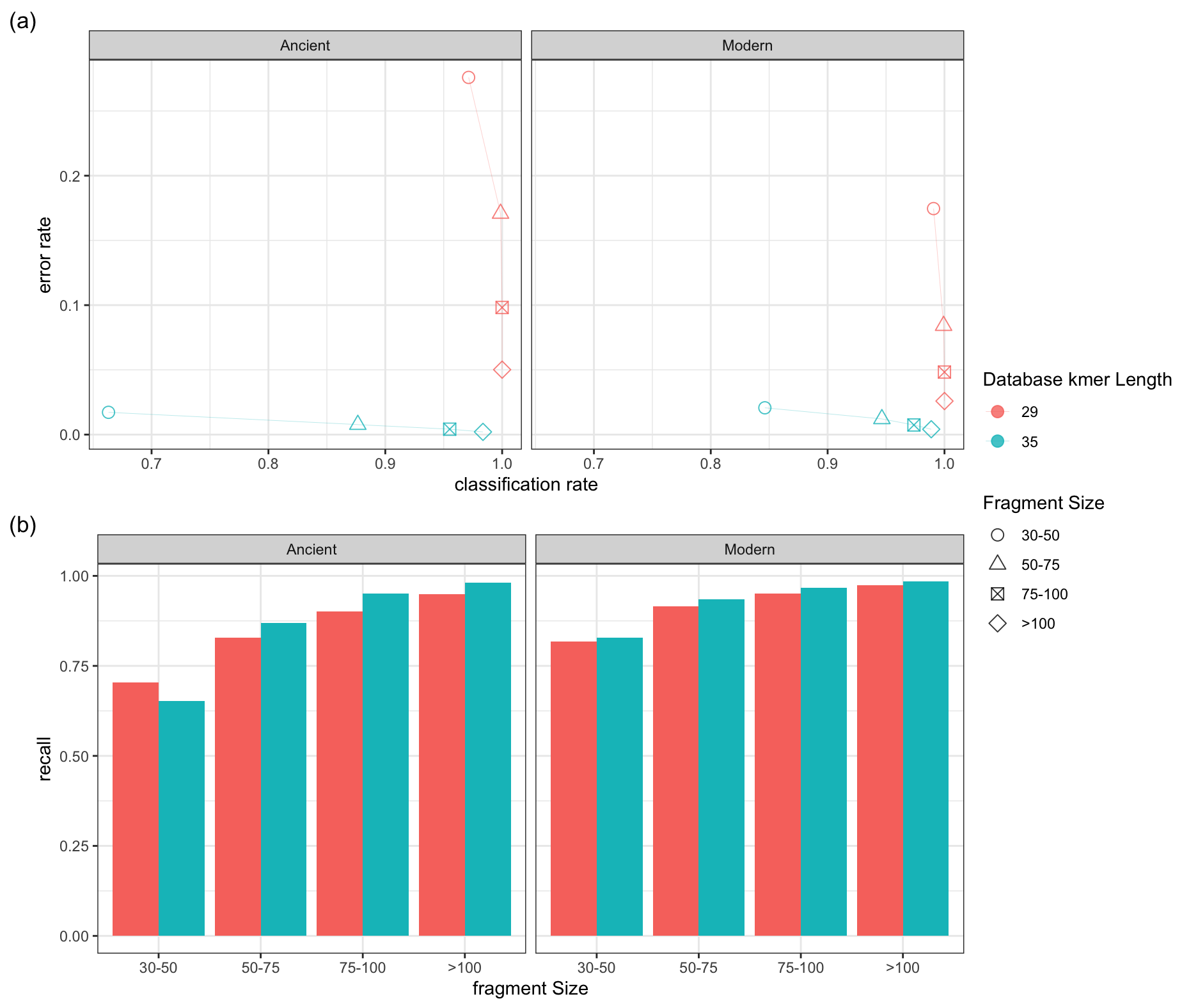
**

**Figure S2:** (A) Rate of false classifications and unclassified reads for ancient and modern human reads classified by the ‘*k2_microbes_human*’ database built with k-mer lengths 29 and 35. Shorter fragments have a higher rate of unclassified reads; (B) Recall of the two databases for different fragment lengths. The k-mer 35 database has similar error rates for ancient and modern reads, indicating deamination does not affect false classifications. The k-mer 29 database, on the other hand, shows a higher error rate for ancient reads, indicating a role of deamination, however, it also has a very high classification rate. Overall, the recall between the two databases is comparable, with the k-mer 29 database showing slightly higher recall for short ancient reads.

*
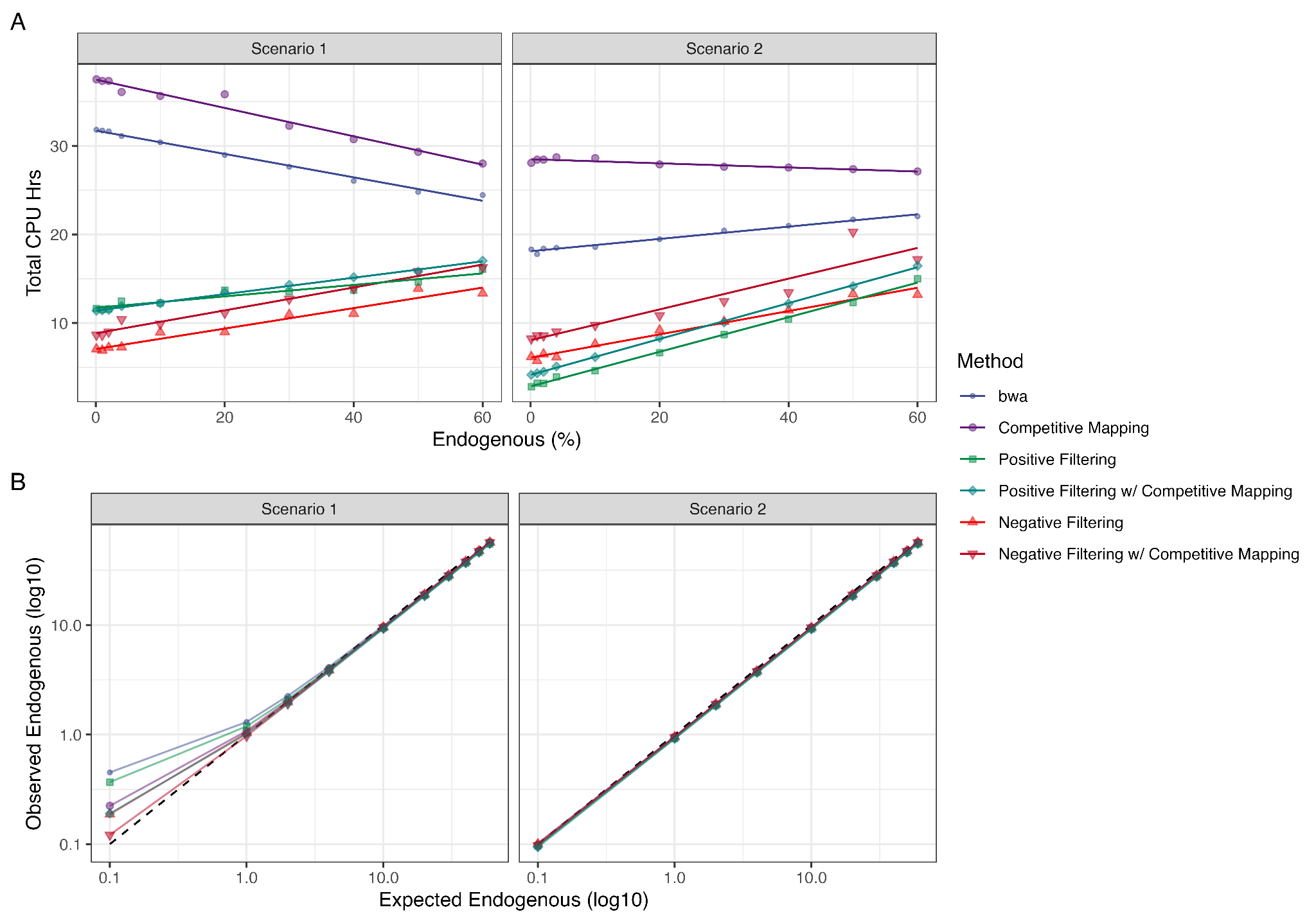
*

**Figure S3:** (A) CPU time to map with bwa aln to single and compositive reference (‘bwa’ and ‘Competitve Mapping’, respectively), postitve and negatative filtering before mapping to a single reference (‘Postitve Filtering’ and ‘Negative Filtering’, respectively), and postitve and negatative filtering before mapping to a compositive reference ( ‘Postitve Filtering w/ Competitive Mapping’ and ‘Negative Filtering w/ Competitive Mapping’, respectively) for dog simulations; (B) Expected and observed endogenous fractions after mapping and filtering with MapQ > 20.


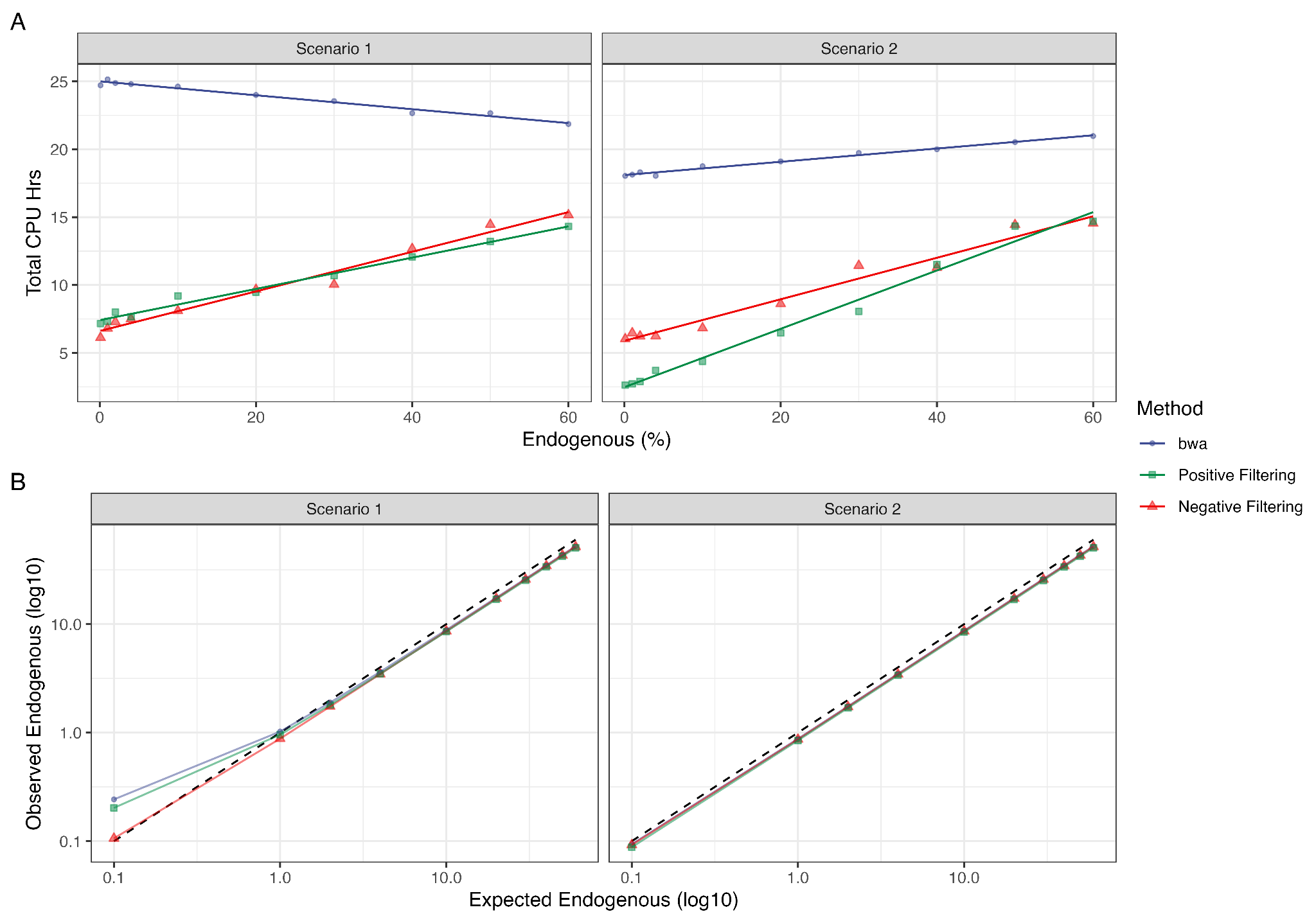


**Figure S4:** (A) CPU time to map with bwa aln to single and compositive reference (‘bwa’), and postitve and negatative filtering before mapping to a single reference (‘Postitve Filtering’ and ‘Negative Filtering’, respectively) for human simulations; (B) Expected and observed endogenous fractions after mapping and filtering with MapQ > 20.


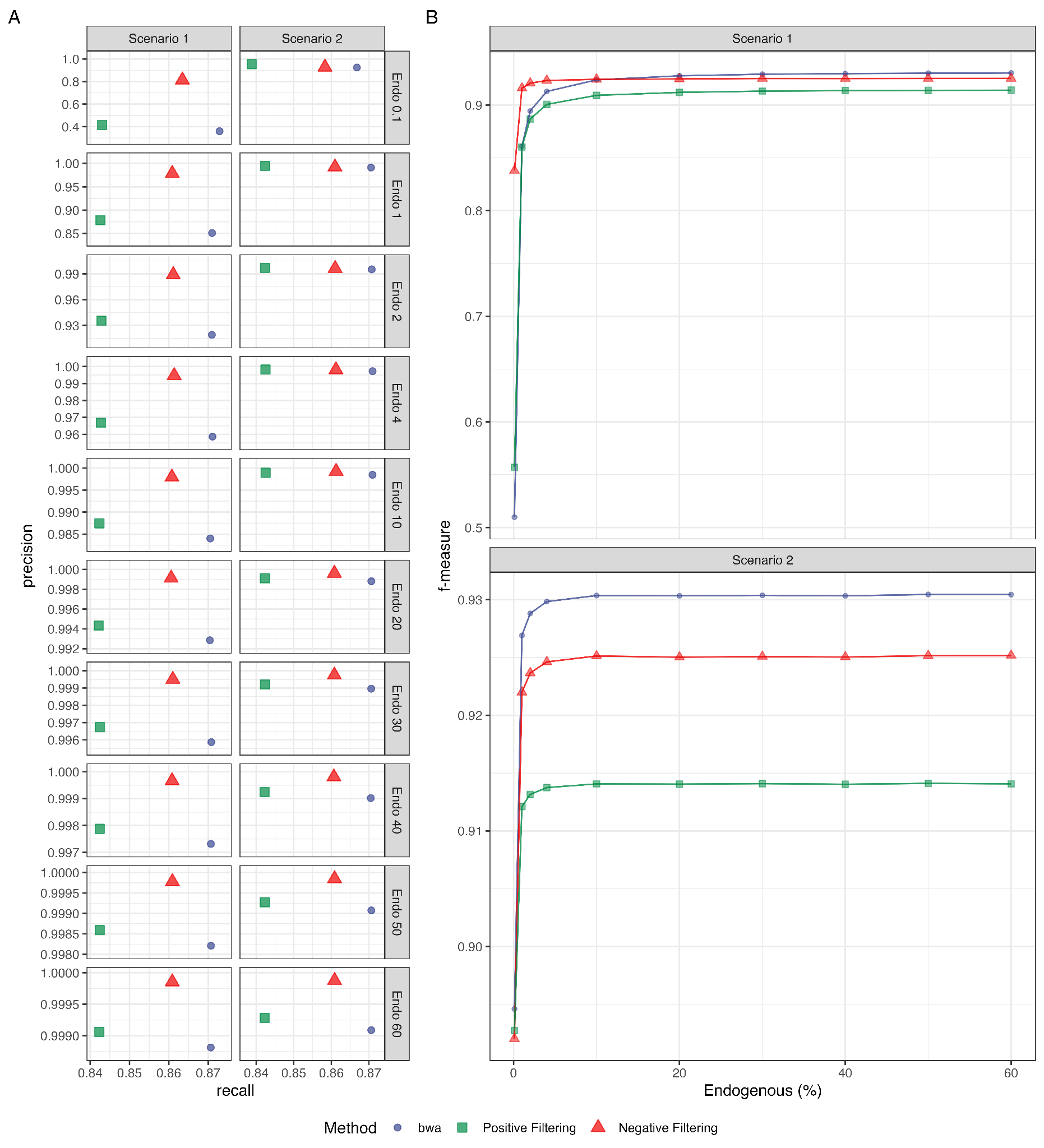


**Figure S5:** Precision and recall (A) and f-measure (B) of the 3 methods: bwa to a single reference (blue circle; ‘bwa’), mapping only reads classified as Primate and unclassified reads by the ‘*k2_custom*’ database (red triangle; ‘Negative Filtering’), and mapping only reads classified by “*k2_human_kmer29*’ database (green square; ‘Positive Filtering’); post mapping and filtering with MapQ > 20 filters for all the simulated ancient dog genome.


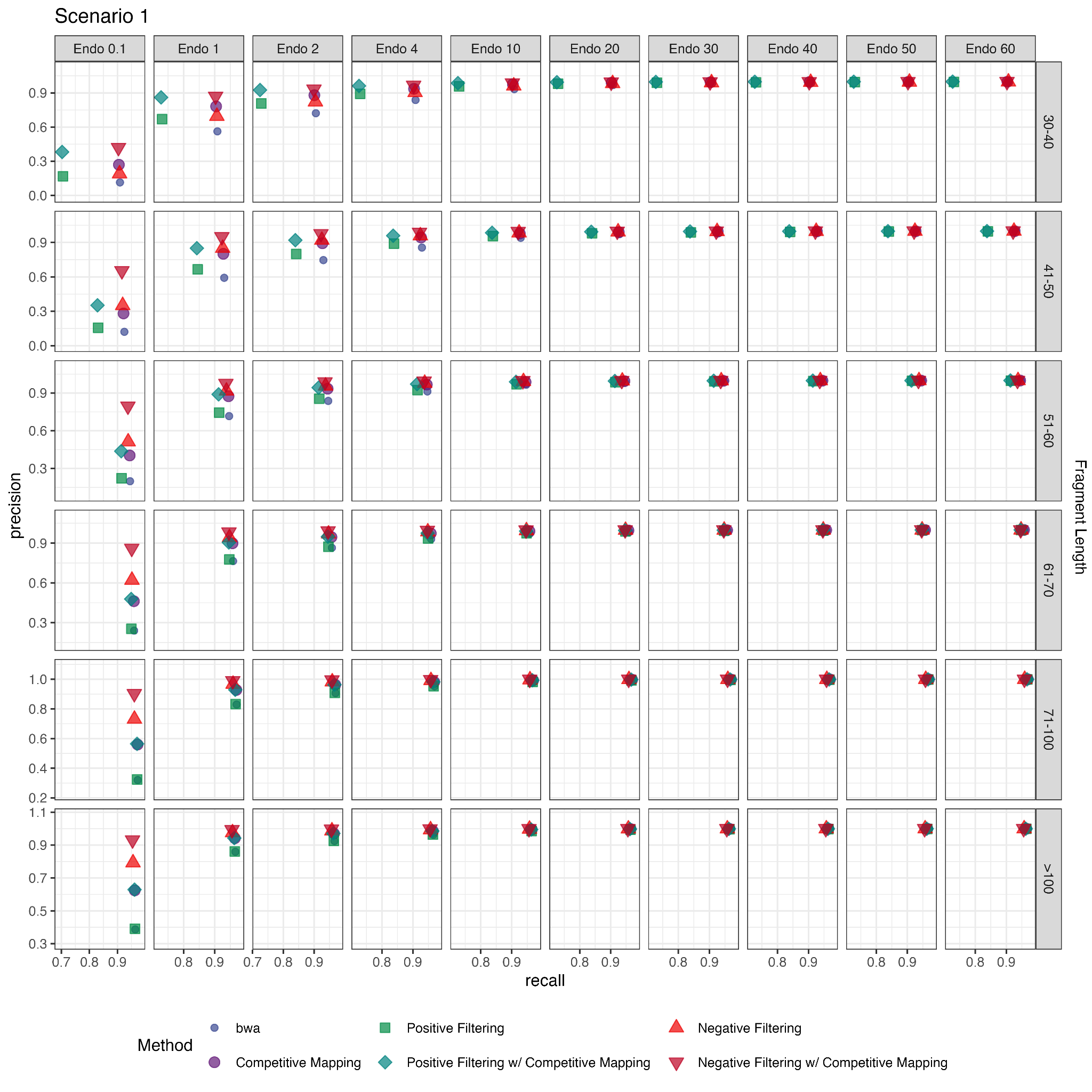
**Figure S6:** Precision and recall of ancient dog genomes calculated across different endogenous fractions and fragment lengths for Scenario 1. The lables represent bwa aln to single and compositive reference (‘bwa’ and ‘Competitve Mapping’, respectively), postitve and negatative filtering before mapping to a single reference (‘Postitve Filtering’ and ‘Negative Filtering’, respectively), and postitve and negatative filtering before mapping to a compositive reference ( ‘Postitve Filtering w/ Competitive Mapping’ and ‘Negative Filtering w/ Competitive Mapping’, respectively).


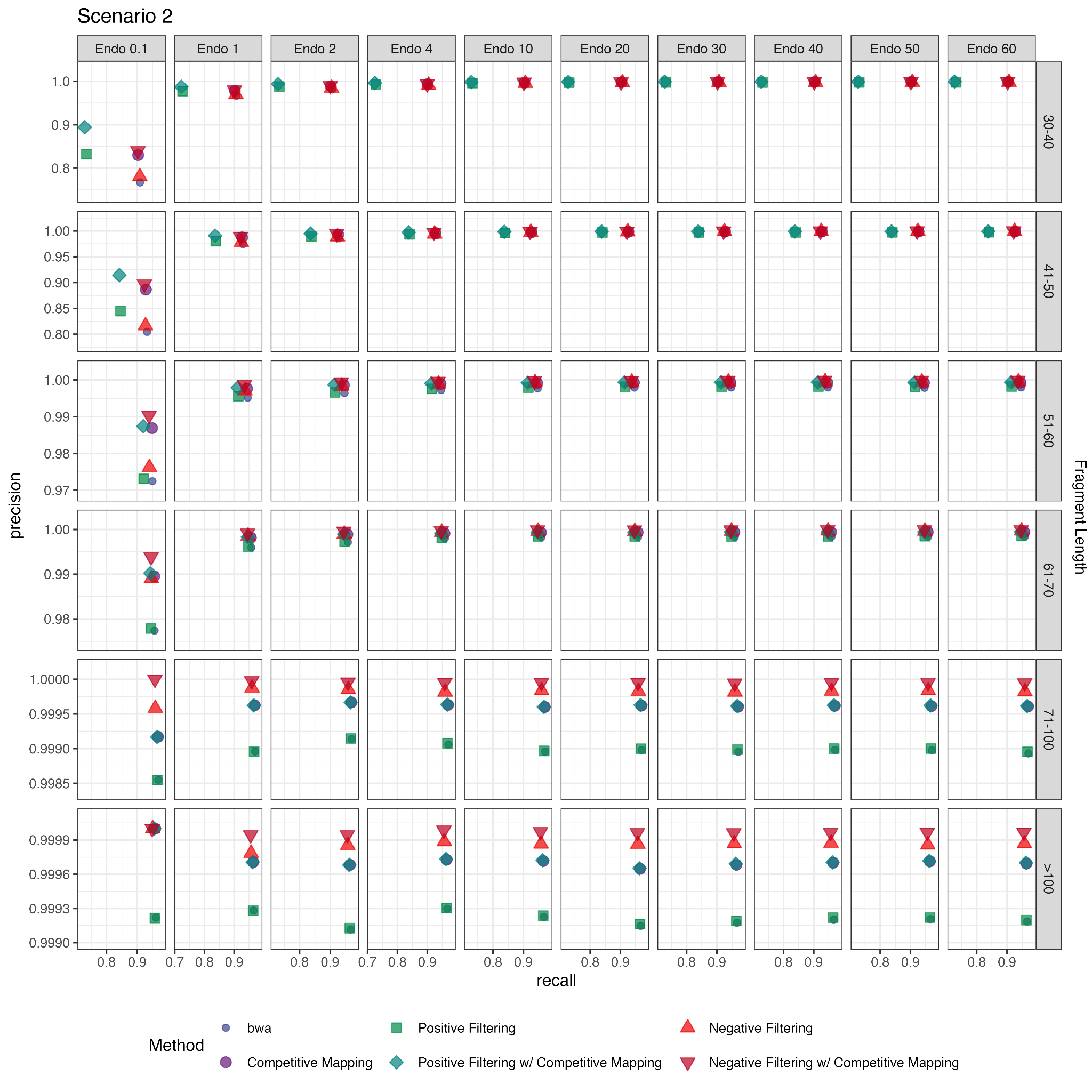
**Figure S7:** Precision and recall of ancient dog genomes calculated across different endogenous fractions and fragment lengths for Scenario 2. The lables represent bwa aln to single and compositive reference (‘bwa’ and ‘Competitve Mapping’, respectively), postitve and negatative filtering before mapping to a single reference (‘Postitve Filtering’ and ‘Negative Filtering’, respectively), and postitve and negatative filtering before mapping to a compositive reference ( ‘Postitve Filtering w/ Competitive Mapping’ and ‘Negative Filtering w/ Competitive Mapping’, respectively).


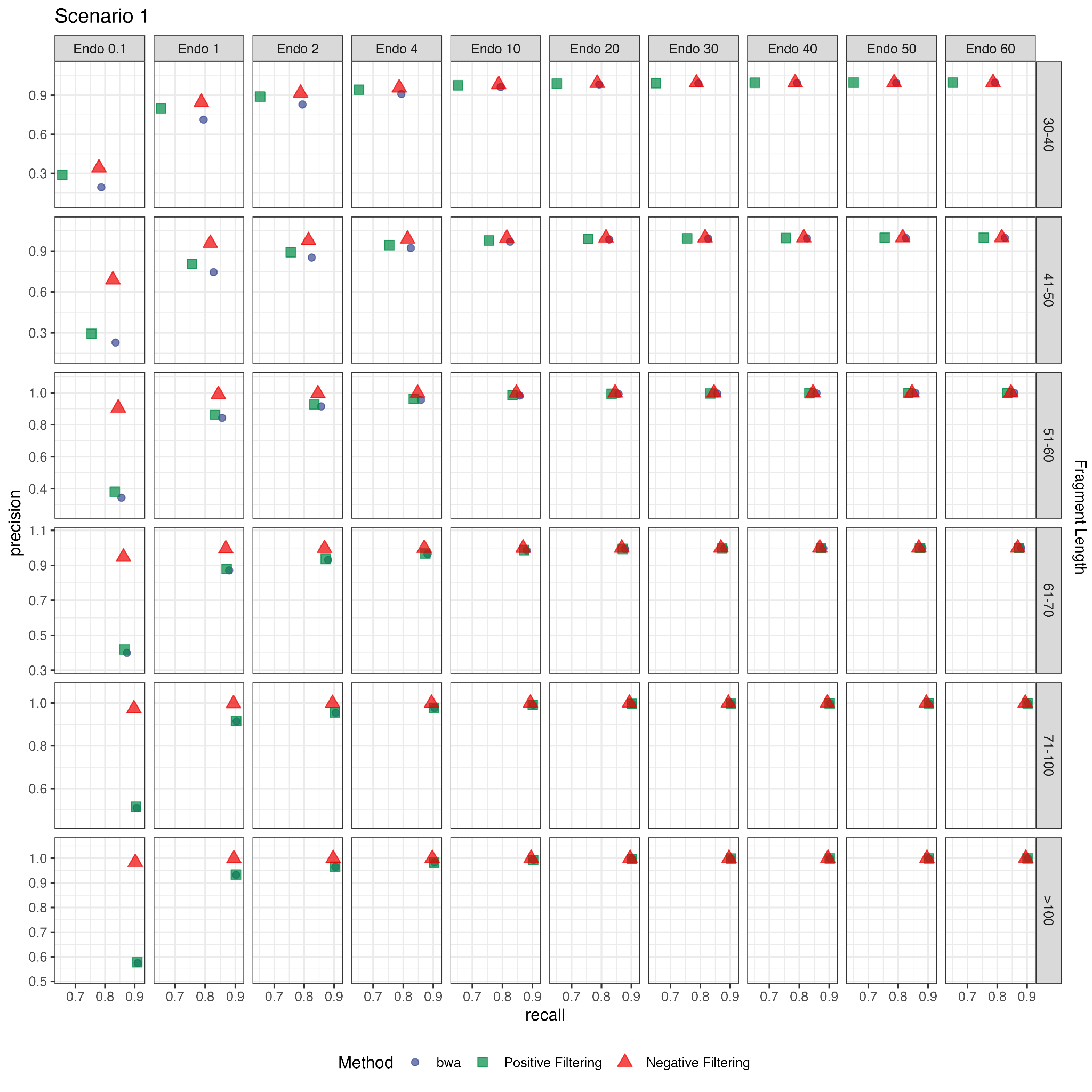


**Figure S8:** Precision and recall of ancient human genomes calculated across different endogenous fractions and fragment lengths for Scenario 1. The lables represent bwa aln to the human referece reference (‘bwa’), postitve and negatative filtering before mapping to the human reference (‘Postitve Filtering’ and ‘Negative Filtering’, respectively).


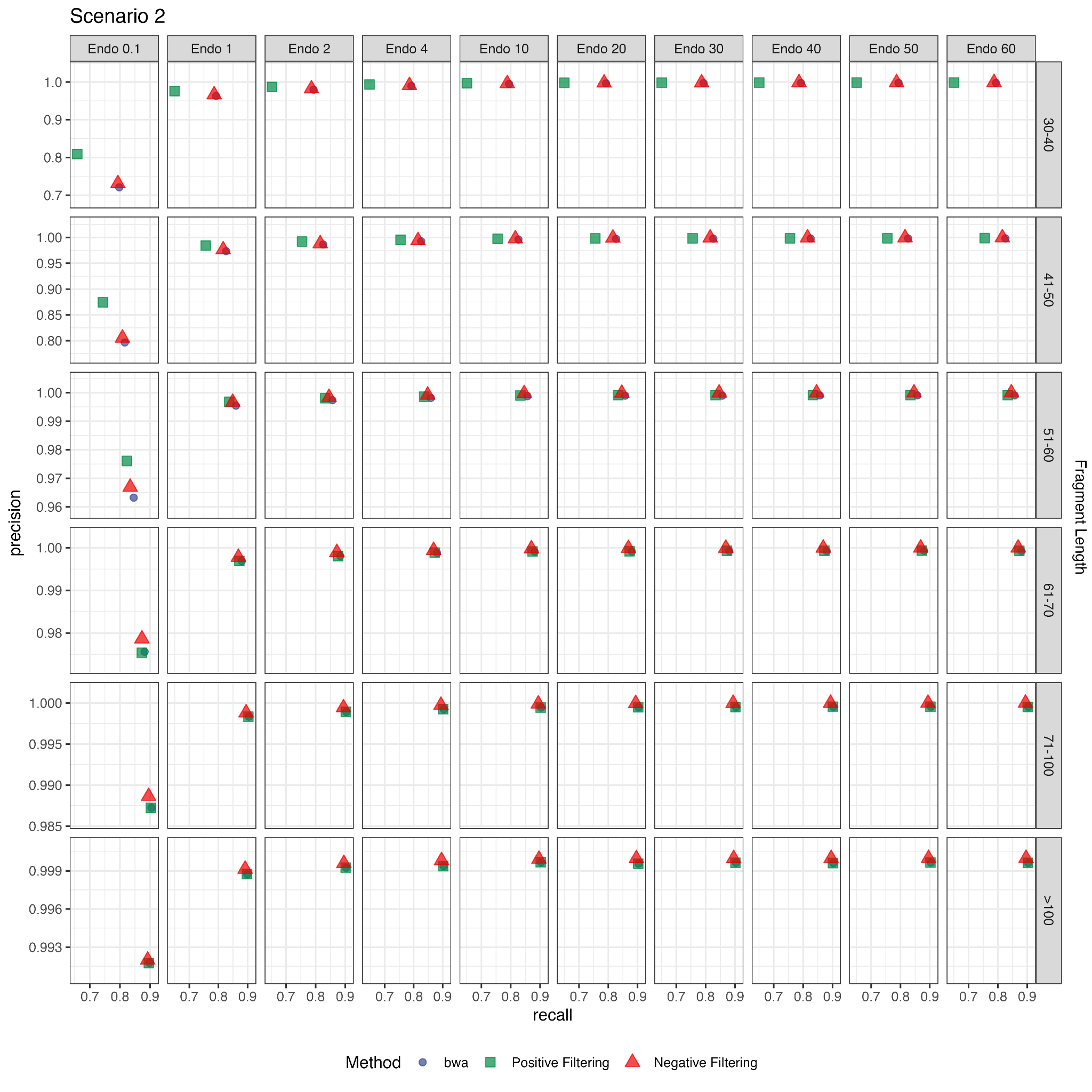


**Figure S9:** Precision and recall of ancient human genomes calculated across different endogenous fractions and fragment lengths for Scenario 1. The lables represent bwa aln to the human referece reference (‘bwa’), postitve and negatative filtering before mapping to the human reference (‘Postitve Filtering’ and ‘Negative Filtering’, respectively).


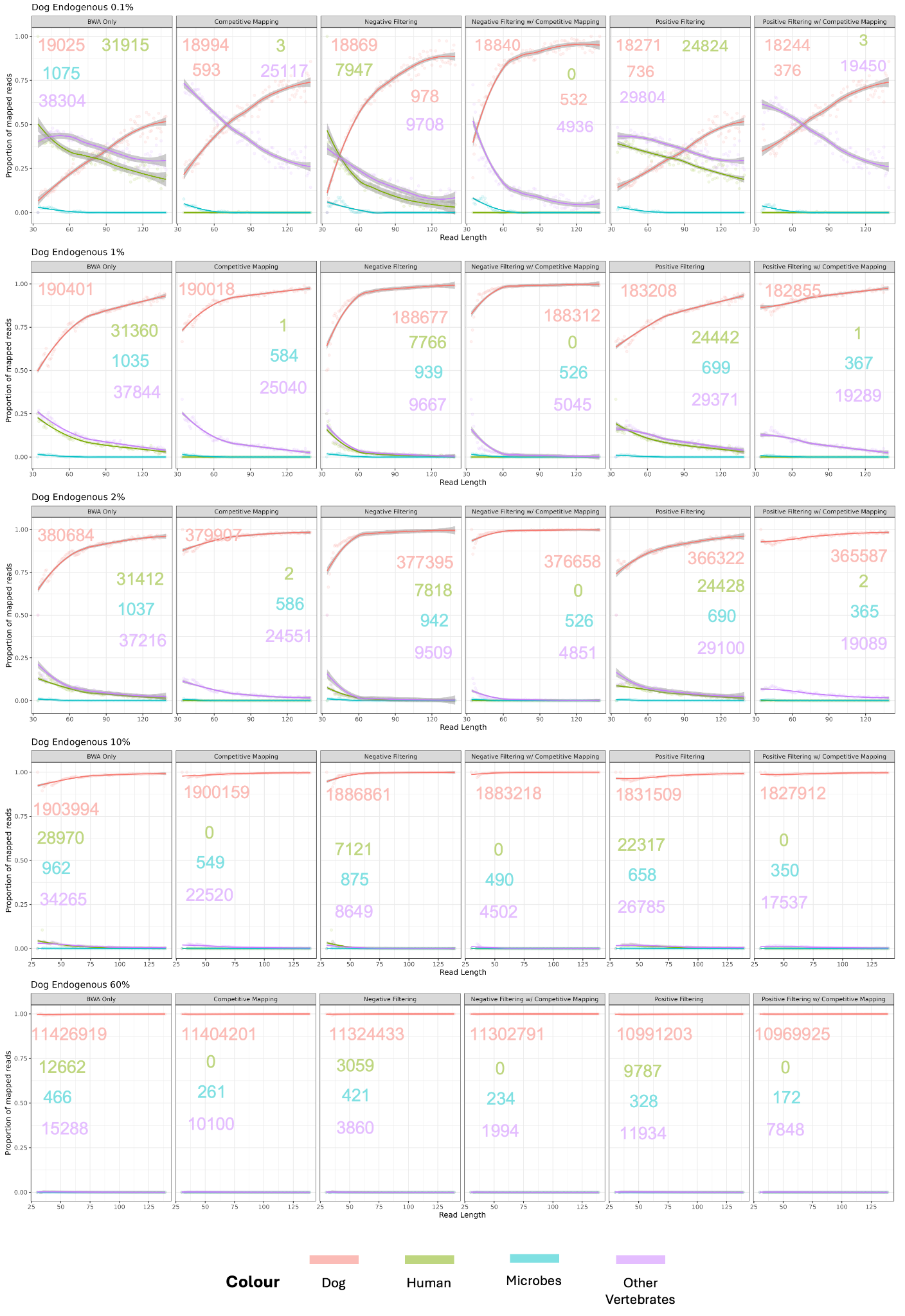


**Figure S10:** Origin of the proportion of reads (colours indicate origin) mapped to the *CanFam6* reference genome using different methods (described in the facets), for ancient dog reads simulated under scenario 1 with high vertebrate contamination. The numbers indicate reads mapped to the reference with mapping quality above 20.


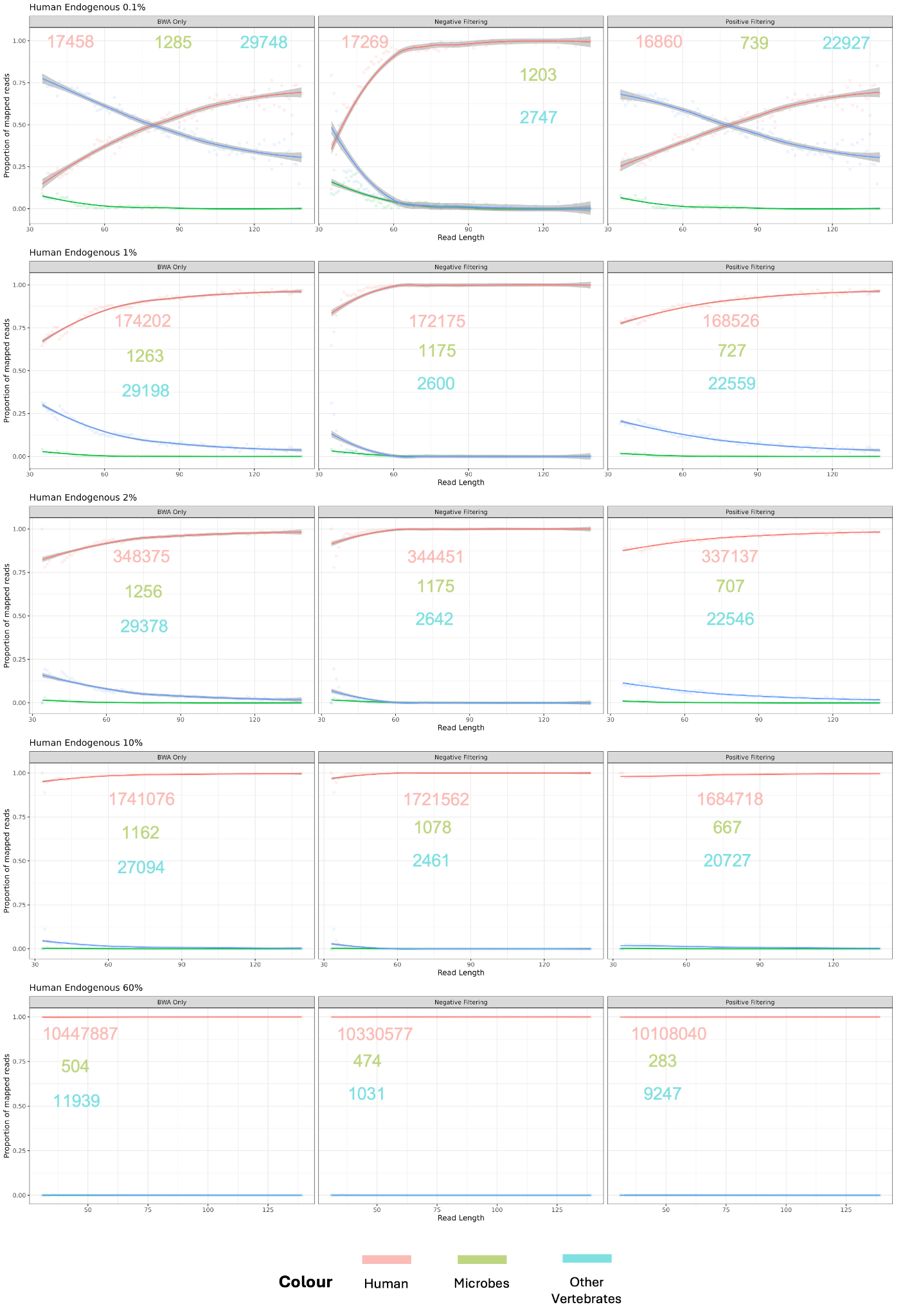


**Figure S11:** Origin of the proportion of reads (colours indicate origin) mapped to the *CanFam6* reference genome using different methods (described in the facets), for ancient dog reads simulated under scenario 2 with lower vertebrate contamination. The numbers indicate reads mapped to the reference with mapping quality above 20.


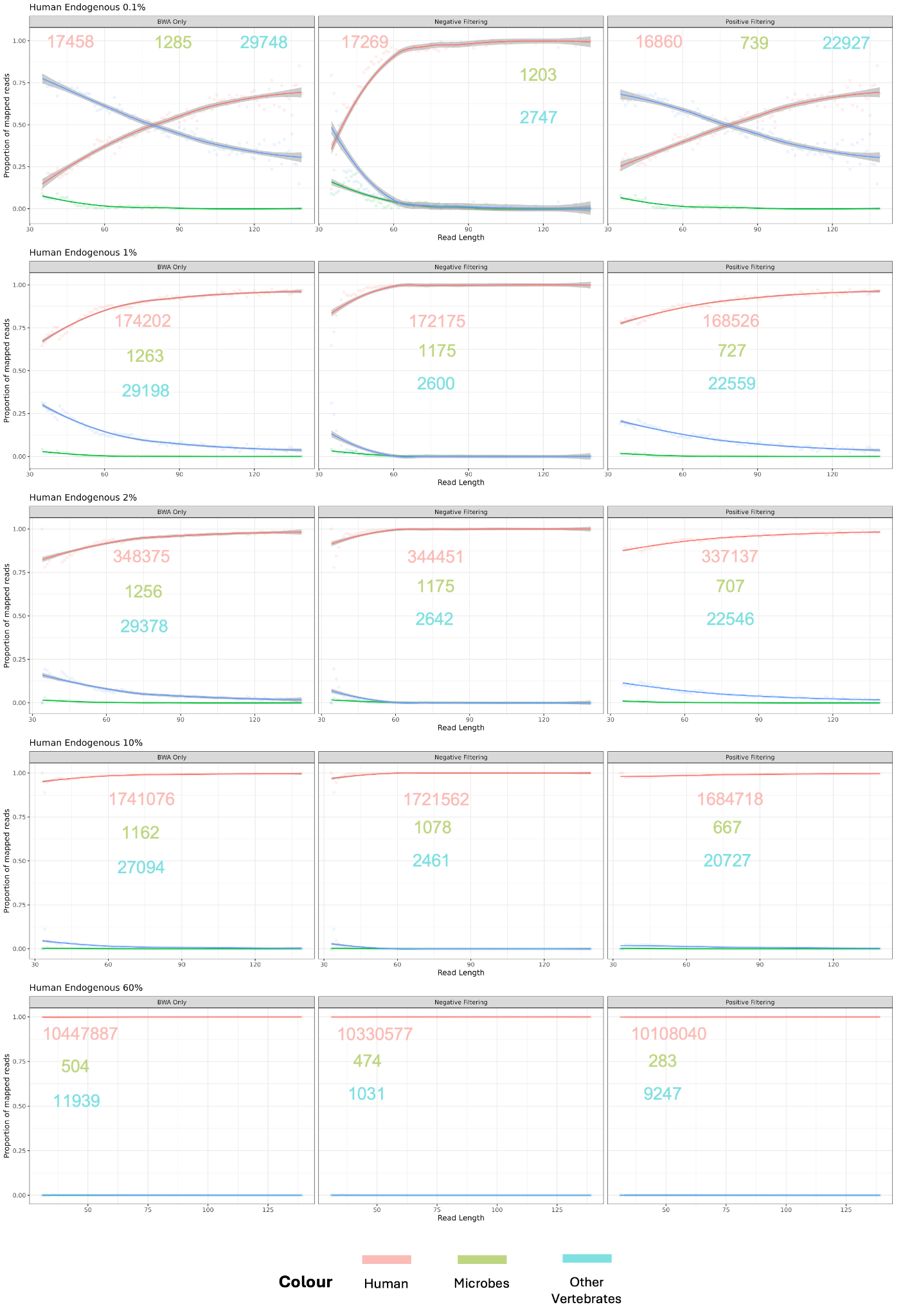


**Figure S12:** Origin of the proportion of reads (colours indicate origin) mapped to the *GRCh38.p14* reference genome using different methods (described in the facets), for ancient human reads simulated under scenario 1 with high vertebrate contamination. The numbers indicate reads mapped to the reference with mapping quality above 20.


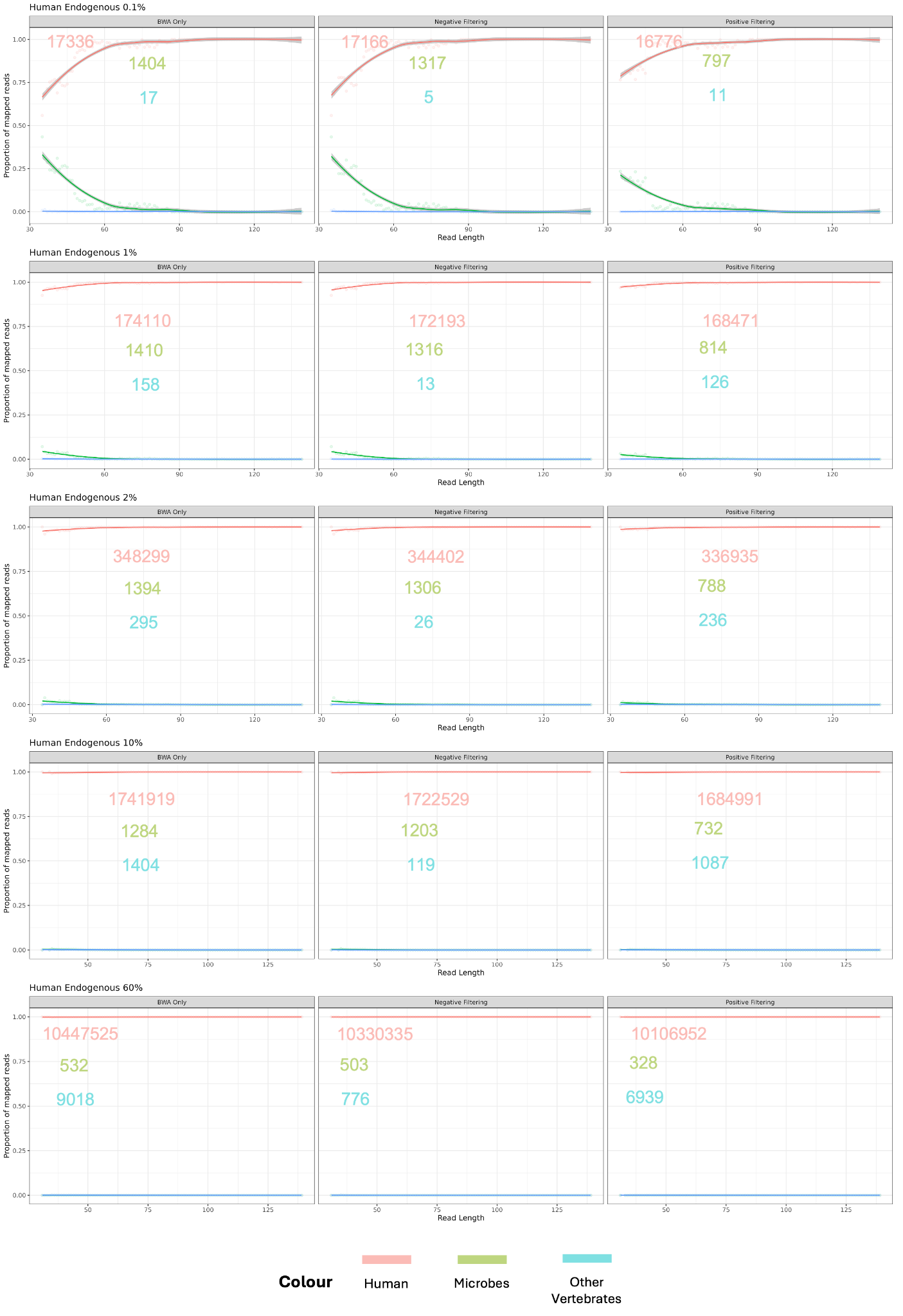


**Figure S13:** Origin of the proportion of reads (colours indicate origin) mapped to the *GRCh38.p14* reference genome using different methods (described in the facets), for ancient human reads simulated under scenario 2 with lower vertebrate contamination. The numbers indicate reads mapped to the reference with mapping quality above 20.


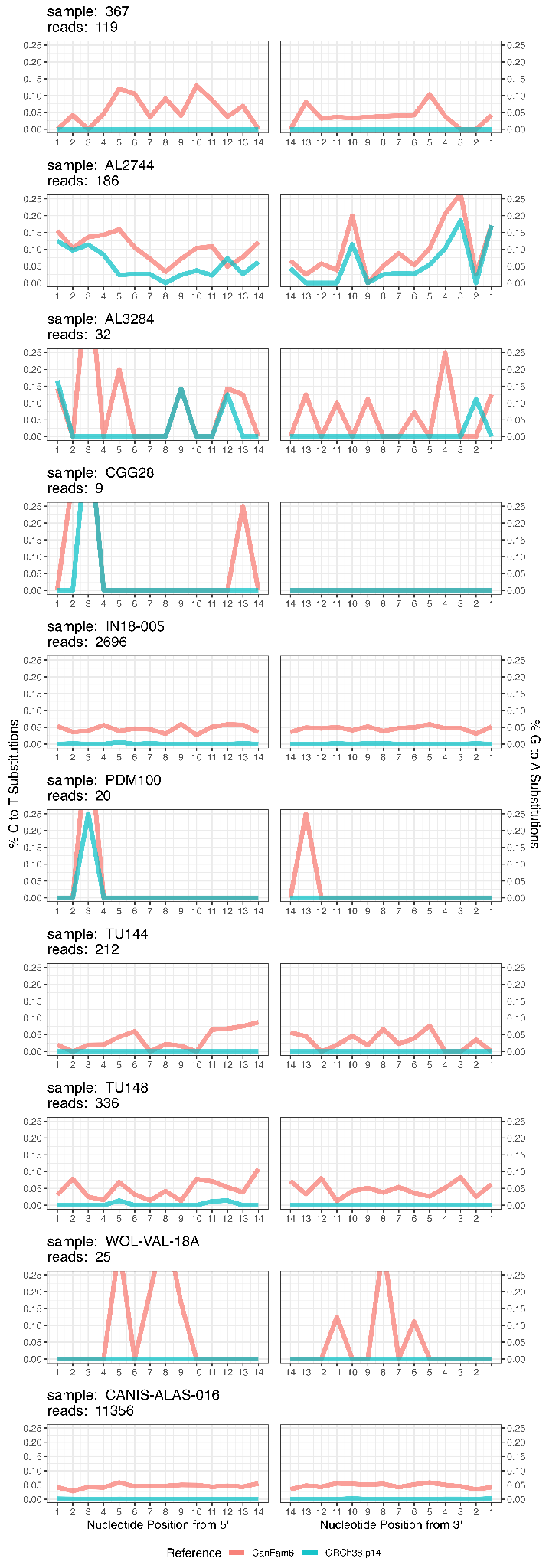


**Figure S14:** Damage profile of reads classified as order Primates by BLAST; mapped to *CanFam6* (red) and *GRCh38.p14* (blue) reference for all ancient wolf samples. These reads were filtered by Kraken2 but mapped to *CanFam6* reference with mapping quality > 20.

## Supplementary Tables

**Table S1**: Breakdown of the read proportions for different endogenous fractions for the two scenarios simulated.

| **Endogenous (%)** | **Scenario 1 Contaminants (%)** | | **Scenario 2 Contaminants (%)** | |
| --- | --- | --- | --- | --- |
|  | **Microbial** | **Vertebrate** | **Microbial** | **Vertebrate** |
| 0.1 | 79.92 | 19.98 | 99.89 | 0.01 |
| 1 | 79.2 | 19.8 | 98.9 | 0.1 |
| 2 | 78.4 | 19.6 | 97.8 | 0.2 |
| 4 | 76.8 | 19.2 | 95.6 | 0.4 |
| 10 | 72 | 18 | 89 | 1 |
| 20 | 64 | 16 | 78 | 2 |
| 30 | 56 | 14 | 67 | 3 |
| 40 | 48 | 12 | 56 | 4 |
| 50 | 40 | 10 | 45 | 5 |
| 60 | 32 | 8 | 34 | 6 |

**Table S2**: Proportions of reads from different sources that are simulated as microbial and reagent contamination.

| **RefSeq Accession** | **Assembly Name** | **Oragnism Name** | **Proportion of reads** |
| --- | --- | --- | --- |
| *Microbial Communities from Seguin-Orlando et al.* | | | |
| GCF_000865745.1 | ViralMultiSegProj15555 | Virus | 0.1850352319 |
| GCF_000856285.1 | ViralProj14970 | Virus | 0.1370056021 |
| GCF_000897015.1 | ViralProj162489 | Virus | 0.1153730119 |
| GCF_000024865.1 | ASM2486v1 | Bacteria | 0.1091923865 |
| GCF_000421765.1 | ASM42176v1 | Bacteria | 0.06386271838 |
| GCF_000850525.1 | ViralProj14756 | Virus | 0.04739656298 |
| GCF_000881515.1 | ViralProj31291 | Mycobacterium phage Cali | 0.03176968244 |
| GCF_000868265.2 | ViralProj17147 | Mycobacterium phage Halo | 0.02645079811 |
| GCF_000910735.1 | ViralProj214366 | Virus | 0.02605763877 |
| GCF_000502975.1 | Mloti_LNJC384A00 | Bacteria | 0.02563318714 |
| GCF_000012745.1 | ASM1274v1 | Bacteria | 0.02485328737 |
| GCF_000372645.1 | Caulobacter.strain_OR37_v1.0 | Bacteria | 0.02043064589 |
| GCF_000013385.1 | ASM1338v1 | Bacteria | 0.01976949629 |
| GCF_000008345.1 | ASM834v1 | Bacteria | 0.01870154712 |
| GCF_000067165.1 | ASM6716v1 | Bacteria | 0.01541906775 |
| GCF_000195775.1 | ASM19577v1 | Bacteria | 0.01540542752 |
| GCF_000847945.1 | ViralProj14585 | Virus | 0.01517033428 |
| GCF_000852985.1 | ViralProj14798 | Virus | 0.01505318884 |
| GCF_000020545.1 | ASM2054v1 |  | 0.01431260501 |
| GCF_000504245.1 | ASM50424v1 | Bacteria | 0.01204431628 |
| GCF_000355695.1 | ASM35569v1 | Bacteria | 0.009519270425 |
| GCF_000212695.1 | ASM21269v1 | Bacteria | 0.009312259992 |
| GCF_000240225.1 | ASM24022v2 | Bacteria | 0.008874970513 |
| GCF_000296405.1 | Turi_otit_ATCC_51513_V1 | Bacteria | 0.008521929466 |
| GCF_000152305.1 | ASM15230v1 | Bacteria | 0.0085211271 |
| GCF_000170395.1 | ASM17039v1 | Bacteria | 0.008169690784 |
| GCF_000309825.1 | ASM30982v2 | Bacteria | 0.008144015072 |
| *Common Reagent Contaminating sources* | | | |
| GCF_016772045.1 | ARS-UI_Ramb_v2.0 | Ovis aries (sheep) | 0.0375 |
| GCF_002263795.2 | ARS-UCD1.3 | Bos taurus (cattle) | 0.8 |
| GCF_000003025.6 | Sscrofa11.1 | Sus scrofa (pig) | 0.0625 |
| GCF_001704415.2 | ARS1.2 | Capra hircus (goat) | 0.0375 |
| GCF_000002315.6 | GRCg6a | Gallus gallus (chicken) | 0.0625 |

**Table S3**: Categorising simulated reads for baseline mapping (bwa)

| **bwa** | | |
| --- | --- | --- |
|  | **Pass MapQ Filter** | **Failed MapQ Filter** |
| **Endogenous Reads** | True Positive | False Negative |
| **Contaminating Reads** | False Positive | True Negative |

**Table S4:** Categorising simulated dog reads mapped with a composite reference (Competitive Mapping)

| **Competitive Mapping** | | | |
| --- | --- | --- | --- |
|  | **Pass MapQ Filter** | **Failed MapQ Filter** | **Mapped to Human Contigs** |
| **Endogenous Reads** | True Positive | False Negative | False Negative |
| **Human Contamination** | False Positive | True Negative | True Negative |
| **Non-human Contamination** | False Positive | True Negative | True Negative |

**Table S5:** Categorising simulated reads with Kraken2 filtering before mapping (Kraken2 + bwa)

| **Kraken2 + bwa** | | | |
| --- | --- | --- | --- |
|  | **Pass Kraken2 Filter** | | **Failed Kraken2 Filter** |
|  | **Pass MapQ Filter** | **Failed MapQ Filter** |  |
| **Endogenous Reads** | True Positive | False Negative | False Negative |
| **Contaminating Reads** | False Positive | True Negative | True Negative |

**Table S6**: Empirical ancient wolf genomes used to test filtering on empirical data

| **Accession ID** | **Sample ID** | **Endogenous DNA content** |
| --- | --- | --- |
| SAMEA13128056 | 367 | 0.40% |
| SAMEA13128101 | PDM100 | 1.50% |
| SAMEA13128093 | TU148 | 4.70% |
| SAMEA13128092 | TU144 | 9.70% |
| SAMEA13128117 | WOL-VAL-18A | 19.20% |
| SAMEA13128122 | AL3284 | 20.80% |
| SAMEA13128076 | CGG28 | 30.50% |
| SAMEA13128107 | CANIS-ALAS-016 | 40.30% |
| SAMEA13128119 | IN18-005 | 48.70% |
| SAMEA13128063 | AL2744 | 61.30% |
